# Effect of green and roasted coffee storage conditions on selected characteristic quality parameters

**DOI:** 10.1038/s41598-023-33609-x

**Published:** 2023-04-20

**Authors:** Justyna Błaszkiewicz, Ewa Nowakowska-Bogdan, Krzysztof Barabosz, Renata Kulesza, Ewa Dresler, Piotr Woszczyński, Łukasz Biłos, Dominika Barbara Matuszek, Krzysztof Szkutnik

**Affiliations:** 1 The Łukasiewicz Research Network-Institute of Heavy Organic Synthesis “Blachownia”, Energetykow 9, 47-225 Kedzierzyn-Kozle, Poland; 2 Mechanical Engineering, Opole University of Technology Doctoral School, Prószkowska 76, 45-758 Opole, Poland; 3 Faculty of Production Engineering and Logistics, Opole University of Technology, Proszkowska 76, 45-758 Opole, Poland; 4 Hard Beans Coffee Roasters Sp. z o.o., Armii Krajowej 35, 45-071 Opole, Poland

**Keywords:** Analytical chemistry, Chemical engineering

## Abstract

The paper attempts to determine the best storage conditions for green and roasted coffee beans. Coffee beans were processed in various ways—some of them were washed or left in their natural state after harvesting, then they were stored in two types of packaging with different permeability, i.e. jute bag and GrainPro polymer bag, ensuring stable conditions in three temperature chambers, i.e. − 10, 10 and 20 °C. The grains treated in this way were evaluated after 3, 6, 9, and 12 months. The selection of the analyzed parameters (in roasted coffee—cupping and 3 selected volatile organic compounds; in green coffee—average water activity, content of volatile fatty acids, 6 selected volatile organic compounds) was to monitor the ongoing processes, and thus the qualitative changes taking place in grains. The research shows that grain stored at 20 °C ages the fastest. Grains stored in − 10 °C and 10 °C chambers perform similarly well. The evaluation of the parameters shows that among the grains stored in these two chambers, the method of grain processing (Natural/Washed) had a greater impact on the results, while the type of packaging did not differentiate the grains to such a significant extent.

## Introduction

For decades, conducted experiments have focused on analysing the quality parameters of coffee beans and ready-to-drink beverages from them^1–3^. Apart from the fact that coffee is the most popular drink consumed, research has been supported by a noticeable upward trend in its consumption in recent years^4^. The growing expectations of commercial customers mean that manufacturers and suppliers face the challenge of constantly raising their standards. Therefore, it is in the interest of coffee producers and importers to provide the best possible conditions for the harvested beans to obtain a high-quality product. It should be noted that although the production of coffee beans is seasonal, their consumption takes place all year round^5^. It is therefore profitable to maintain the parameters of the harvested coffee at a stable, high level, which will transform into a high commercial value of the product.

The factors that influence the quality of the beans can be defined as pre- and post-harvest^6,7^. In this study, we focused on and monitored post-collective factors.

The process of obtaining ready-to-brew coffee beans is long and multi-stage. The processing chain begins with the initial seed preparation. Three methods are commonly used to remove the outer layers of the grain (rind and flesh)^5,8^. In the first one,- dry (natural) processing seeds are exposed to the sun or air dryers until the desired moisture level is achieved. After drying, the fruit is cleaned and peeled, then the outer layer of the grain is removed. Wet treatment looks roughly different, as it involves a complex series of steps including the mechanical removal of the crust and coffee pulp, the microbial degradation (fermentation) of the mucus layer, and finally the removal of water by sun drying. It is worth mentioning that the latter procedure significantly shortens the drying time. Compared to dry processing, which takes about 3–5 weeks, the wet version is only 8–10 days. An intermediate treatment is a semi-dry process, which includes the steps of both dry and wet methods, in which the coffee fruit is mechanically removed from the pulp and then sun-dried^9^. The selection of the drying method depends on the economy, but more importantly on environmental factors (latitude where coffee bushes are grown). The process of preparing coffee beans should be started as soon as possible to prevent unwanted fermentation or mold formation^10^. The key aspect is the final drying of the beans to 10–12% of moisture, which will not allow taking place the above-mentioned unfavorable processes.

Another important factor is the influence of the type of used packaging on the final product attributes. It has also been extensively researched^11–14^. The two main packaging used are jute bags and GrainPro polymer ones. The main difference is that jute bags are not a tight barrier and the grains stored in them are not separated from external factors such as moisture or air. GrainPro bags are made of an impermeable polymer material that allows the test material to be separated from the environment. According to the literature, storing grain in classic jute packaging gives better results of the content of volatile organic compounds than hermetic (vacuum) packaging. This is explained as a consequence of the removal of the volatile substances present in the packaging in the air. At the same time, as expected, the perforated classic bags had greater losses of the discussed compounds than the non-perforated ones. This is possibly related to the “escape” or oxidation of the compounds. It is worth noting that although the VOC content decreases when the grains are closed, vacuum packaging is allowed to maintain the amount of these compounds during storage at an almost unchanged level, which is due to the appropriate impermeability of the packaging.

The last aspect that will be discussed in this publication is the time and temperature in which coffee will be stored. Many chemical, biological, and physical changes occur during storage and are known as aging. It has an undeniable impact on the quality parameters. Loss of aroma and taste due to lipid oxidation as well as the breakdown of some fragrances over time^15,16^ are occurring then. The GC–MS method, which has been used for many years, is suitable for monitoring the aging process by observing changes in VOCs concentrations that correspond to processes triggered by external factors^17–21^.

## Materials and methods

### Coffee samples preparation

- Washed Arabica coffee: Arabica (*Coffea arabica L.*) green coffee beans were delivered from Finca La Maravilla (1700 masl). The CB was transported from the farm in the town of Libertad to Huehuetenango city (2000 masl.), where it was stored in a dry warehouse on parchment. When the coffee was ready for export it was transported to a dry mill for preparation and processing. After milling, it was packed into GrainPro and jute bags. Coffee samples reached Poland by sea.
- Natural Arabica coffee: Arabica (*Coffea arabica L.*) green coffee beans were delivered from Finca El Oregano (1700 masl). After the coffee was picked it emerged in water to separate the floaters and then it was placed directly on the 3-level raised beds. First 3–5 days on the top level, the next 3–5 days middle level, and the last days lowest level also for 3–5 days. The drying depends greatly on the temperature. It takes 10–15 days due to the dry conditions characteristic of this climate. After the coffee reached 11% moisture it was transported directly into storage from the farm in Huehuetenango city where it was stored on pallets, in jute and GrainPro bags. These coffee beans also arrived in Poland by sea.

### Types of packaging

- GrainPro bag is made of multi-layer PE with a barrier coating. The manufacturer describes^22^ it as hermetic packaging, able to reuse. It can protect dry agricultural commodities against the growth of mould and insects, maintains the germination and vigour of stored seeds, as well as preserves their taste, and colour.
- Castanhal jute bag for coffee is made according to the International Coffee Organization (ICO) standards^23^. It is made from biodegradable natural, organic fiber cultivated in total integration with the Amazon biome. Unlike plastic packaging, jute bags are permeable to water and gases.

### Storage

Samples of green and roasted coffee beans after delivery were placed in controlled temperature conditions, respectively: − 10, 10, and 20 °C. Next, the CB was ground by grinding in a cryogenic mill for the determination of volatile organic compounds. The ground samples were weighed into tightly closed vials/ampoules and stored under controlled temperature conditions until the determination was performed. Samples for the remaining determinations were ground with dry ice. This part of the ground coffee was placed in polyethylene string bags and stored in controlled temperature conditions until the determinations were made. This procedure was repeated for each storage period.

### Roasting

After respectively 3, 6, 9, and 12 months period of storage, green coffee bean samples were removed from each measured chamber for stabilization.

Half were used for further chemical analysis, while the other half was roasted after a day of stabilization.

A ROEST S100 sample roaster designed for optimal workflow and efficiency in a coffee lab maintained the following conditions: roasting start temperature: 165 °C, final temperature: 205 °C, roasting time: 6–7 min with a fixed airflow and heater setting, and development time: 53 s.

The heat application during roasting was the same for each sample in each stage of the roasting process.

Moreover, the time from the first crack until the end of the roasting (development) was the same (53 s).

The colour check was performed using a Probat Colorette 4measuring device. Then, the roasted coffee beans were subjected to sensory and chemical analyses.

### Sensory analysis

This analysis was carried out based on the Specialty Coffee Association of America (SCAA) protocol^24^. The green coffee samples were roasted after each storage period for 48 h before cupping. Samples were ground immediately before cupping, no more than 15 min. before infusion with water. The hot water (97 °C) was poured directly into the measured grounds to the rim of the cup. The steeping time took 4 min. before the evaluation process started. Five certified judges in two independent blind cupping tests evaluated seven sensorial attributes (aroma, flavor, aftertaste, acidity, body, balance, and overall impression). Through cupping, it is possible to quantify the quality of the tested beverages. The tested characteristics, i.e. aroma, taste, aftertaste, acidity, texture, balance, and overall rating were judged from 6 to 10 on a 16-point scale that represents the quality level in quarter-point increments, where 6.00–6,75 is good coffee, 7.00–7.75 is very good, 8.00–8.75 is excellent, and when the score is 9.00–9.75 it is considered outstanding. The final grade is calculated by summing up the individual scores given for each aspect, plus clean cup, uniformity, and sweetness.

### Volatile organic compounds analysis

VOCs were determined by a targeted analysis performed by gas chromatography with tandem mass spectrometry (GC–MS/MS). The analytes were isolated by micro-extraction to the stationary phase (SPME) using the fiber composed of the adsorbents: polydivinolbenzene, Carboxen, and polydimethylsiloxane (2 cm, DVB/CAR/PDMS). The extraction parameters were kept: 1 g of the grounded coffee weighed into 20 ml vials, was added the internal standard to the sample, and seal the vial tightly with a Teflon-silicone cap. Extraction was performed at 60 °C for 30 min. The analysis was performed on Agilent Technologies 7890A system with an MSD detector 7000 GC/MS Triple Quad and computer program “MassHunter” with Capillary chromatography column HP-5MS (30 m × 0.25 mm × 0.25 μm) using helium as carrier gas (flow 1 mL/min). The oven temperature program started with a 3-min isotherm of 50 °C, and then, the temperature was increased by 10 °C/min up to 240 °C, which was maintained for 8 min. Mass spectra were acquired in electron impact (EI) mode at 70 eV. Each analysis was carried out every 3 months in triple repetitions (n = 3).

### Free fatty acids analysis

The tests were carried out by gas chromatography with a flame ionization detector (GC-FID). The analysis was performed on A 6890N Agilent Technologies gas chromatograph equipped with an autosampler, a flame ionization detector, and a ZB-5HT column (15 m × 0.25 mm × 0.10 μm). Nonadecanoic acid was added to the ground coffee as an internal standard, and 20 ml of a solution of dichloromethane: methanol (DCM: MeOH 2:1 v/v) was added. The solution was decanted and filtered. Another DCM: MeOH solution was added to the filtrate and filtered. The combined filtrates were evaporated at 50 °C and 337 mbar pressure until ca. 1 ml of extract was obtained. 5 ml of a solution of boron trifluoride (BCl_3_) in methanol (10%) was added to the extract thus obtained and heated for 5 min at 90 °C. Based on the experience in the determination of FFAs, slight changes were made to the methodology of sample preparation. Due to the high concentration of acids in the grain samples and for practical reasons, 20 ml of DCM: MeOH solution was added instead of 10 ml, and the obtained extracts were evaporated under reduced pressure, a solution of boron trifluoride with a concentration of 10% and not 14% was used and the heating time was extended by 3 min. up to 5 min. Each analysis was carried out every 3 months in triple (n = 3).

### Water activity analysis

The research was carried out using the relative method of measuring the water vapour pressure every 3 months, with 5 repetitions (n = 5).

**Figure 1:**
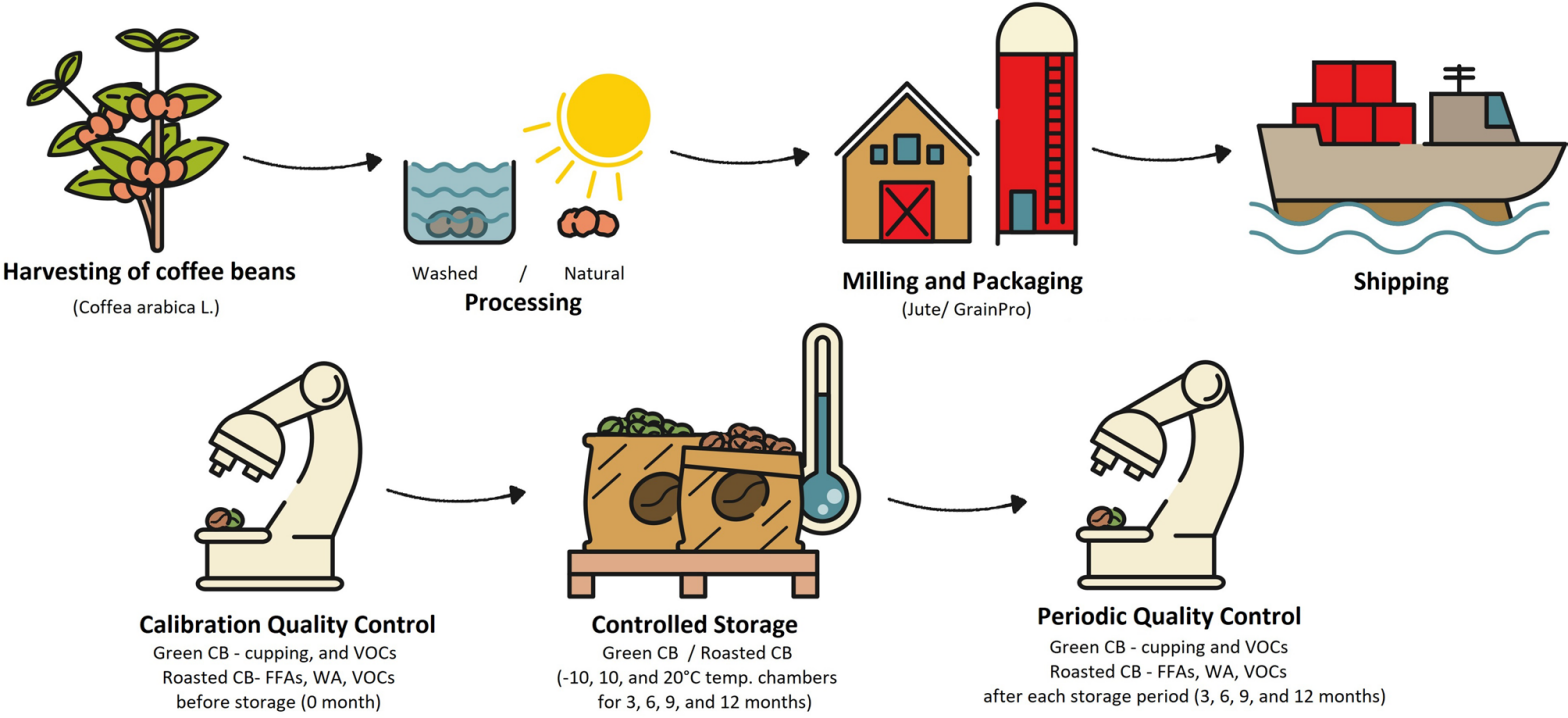
Illustration showing the process that the coffee beans have gone through from picking to analysis.

All the discussed stages are shown in Fig. 1.

### Statistical analysis

For the sensory analysis, the results from 5 cuppers in 2 independent cuppings were used and expressed as the mean (n = 10) ± standard deviation. Mean values were compared using the ANOVA test. Classification techniques such as cluster analysis (CA; Ward tree diagram with Euclidean distance) and principal component analysis (PCA) were used to interpret the obtained results. Calculations were performed using the software Statistica ver. 10. (www.statsoft.pl). A correlation matrix was used to find a significant correlation between the considered variables. Differences were considered significant when the p-value was < 0.05.

### Chemicals and reagents

Analytical standards of methional (CAS: 3268-49-3), 2-Isobutyl-3-Methoxypyrazine (CAS: 24683-00-9), 5-Methylfurfural (CAS: 620-02-0), Methyl 3-Methylbutanoate (CAS: 556-24-1), Ethyl 2-Methylbutyrate (CAS:7452-79-1), Cyclohexanone (CAS: 108-94-1), (E)-2-Nonenal (CAS: 18829-56-6), Hexanal (CAS: 66-25-1), 2-Methoxy-4-vinylphenol (CAS: 7786-61-0), Dimethyl Sulfide (CAS: 75-18-3) were purchased from Chempur**,** Sigma, Alpha Aesar, Fluka, Chromadex, Stanlab, Fisher, TCl. All standards used were of analytical grade (≥ 99% purity). All other chemicals and solvents were of analytical grade.

## Results and discussion

### The influence of storage conditions on sensory evaluation

**Figure 2:**
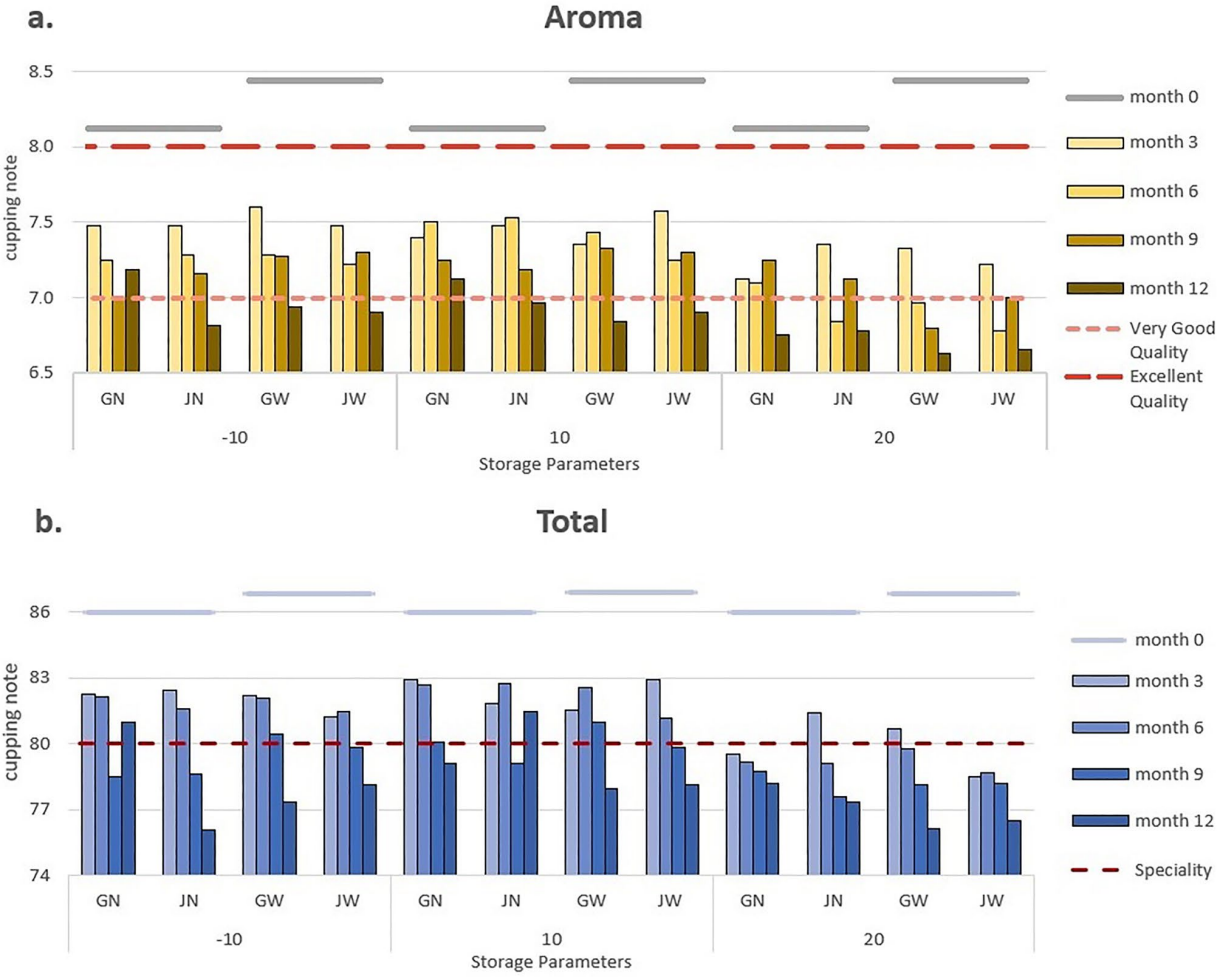
Results of elements of the sensory evaluation for 2(**a**) Aroma and 2(**b**) Total note in the initial roasted coffee storage in − 10, 10, and 20 °C after storage for 3, 6, 9 and 12 months.

As mentioned, cupping is a useful and reliable tool for evaluating coffee quality. Using publicly available guidelines, the judges decide on the note, and thus on the rank that a given drink can boast of Fig. 2 shows, according to the authors, the most important assessments that make up the cupping result (Other charts parameters to be found in the Supplementary).

For all tested parameters noticed a clear downward trend of the grades awarded over time, especially for grains kept in the 20 °C chambers. The greatest decrease was observed after the first three months of storage of the grains. In the examined cases, a gradual downward trend in the cupping assessment of Aroma (Fig. 2a) was observed. It is worth noting that grains stored in chambers − 10 °C and 10 °C were able to receive a score above 7 points (it can be said that they are of very good quality) until the 9th month of storage. In addition, **GN** values from the previously mentioned chambers also managed to stay above 7 points in the 12th month of storage.

The graph in Fig. 2b shows the final (total) score, which is the sum of the individual scores given for each attribute plus sweetness, clean cup, and uniformity. The final score allows you to qualify the coffee as specialty coffee when it exceeds 80 points. Coffees stored in chambers − 10 °C and 10 °C received notes qualifying them as specialty coffees in the initial assessment, after 3 and after 6 months of storage, and the notes after 9 months are in several cases (− 10**GW**, − 10**GW**, 10**GN**, 10**GW**, 10**GW**) are close to 80 points.

The results presented above indicate that the storage of grain, especially after the **W** treatment, in both **J** and **G** bags at a temperature of 10 °C, allows for their long, even 9-month storage, while maintaining relatively good evaluations of cupping parameters.

### The influence of storage conditions on changes in VOCs quantity of roasted grain

Three volatile organic compounds were selected to monitor the changes taking place in the roasted grains.

The first of them, Methional, contains an aldehyde and a thioether group in its structure. Its presence in coffee beans is the result of reactions taking place due to the presence of oxygen during the process of bean storage^7^. During the sensory evaluation, it was found to smell like boiled potatoes^25^.

Another 5-Methylfurfural compound belongs to the group of aldehydes. Its presence in grains is also related to the oxygen atmosphere that prevails during their storage, but also to the wet processing of grains^7^. The team of X. Wang et al.^26^ showed that several VOCs, including the tested furfural, are associated with the deterioration of the quality of roasted coffee during storage. This compound is attributed to a caramel fragrance.

The last of the monitored compounds is 2-Isopropyl-3-Methoxypyrazine. It has been identified as responsible for the negative (earthy) aromatic notes in coffee^7^. Pyrazines and pyridines are formed mainly as a result of the Maillard reaction of amino acids and sugars, direct pyrolysis of amino acids, and degradation of trigonelline^27^. According to Czerny M. and Grosch W.^28^ methoxypyrazines did not reduce their concentration in time, so it seems likely that they can significantly affect the aroma of the coffee drink.

**Figure 3:**
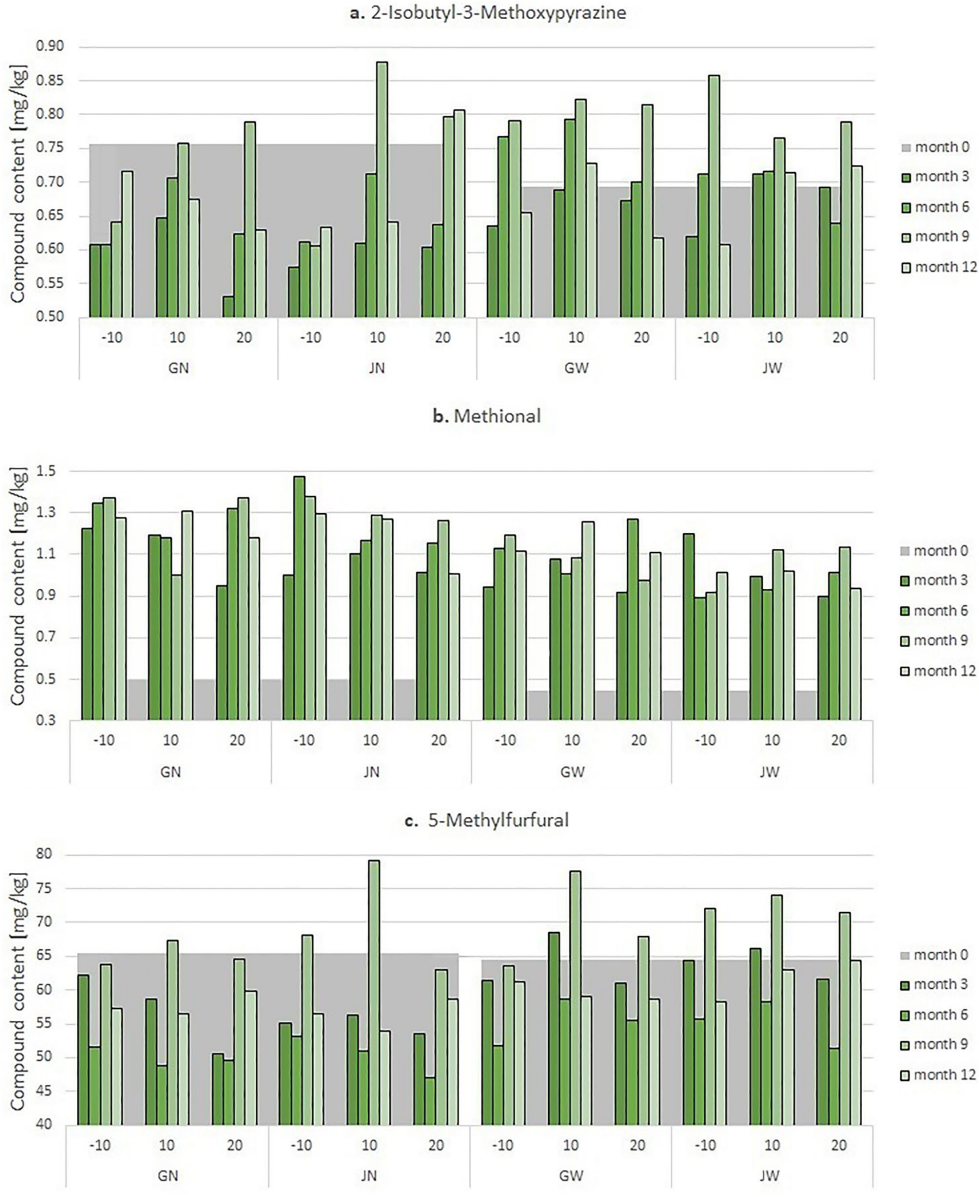
Graph of changes of 2-Isopropyl-3-Methoxypyrazine content of 2-Isobutyl-3-Methoxypyrazine in tested roasted coffee samples on a 12-month basis (with results recorded every 3 months), after appropriate treatment in packaging.

Undeniably, the presence of 2-Isopropyl-3-Methoxypyrazine (Fig. 3) influences the coffee aroma^28,29^, The concentration of this compound is at a rather stable level, although there is a tendency to increase the concentration in the 9th month of grain storage, regardless of the conditions used. This is inconsistent with the information found^30^.

It was found that for Methional (chart presented in Fig. 2 in Supplementary), **W** grains stored in **J** bags were characterized by the highest stability of the concentration. The results collected for all types of beans and bags stored in the 10 °C chamber were the closest. Over the months, Methional concentration rather tended to increase, which confirms the occurrence of processes involving oxygen in grains.

Another volatile organic compound tested in roasted grains, a representative of aldehydes, 5-Methylfurfural, (Fig. 2 in Supplementary) also has a large impact on the aromatic composition^26^, Again, as in the case of 2-Isopropyl-3-Methoxypyrazine there was a tendency to increase the concentration of this compound between the 3rd and 9th month of storage when the concentrations significantly increased above the initial values.

Figure 3 presents a graph of the concentration of 2-Isopropyl-3-Methoxypyrazine in terms of the dry mass of coffee. Graphs showing the concentrations of Methional and 5-Methylfurfural are included in the Supplementary (Fig. 3 in Supplementary).

The observed course of the graphs (Fig. 3) showing changes in the content of the observed VOCs in roasted coffee beans suggests that the recorded changes are more related to the processing method (**N** or **W**) than to the packaging used (**J** or **G**). As can be seen in the figure above, in **W** grains, despite the lower value in initial month 0, grains record rather higher contents of 2-Isobutyl-3-Methoxypyrazine in subsequent months of storage than grains treated in **N** way.

**Figure 4:**
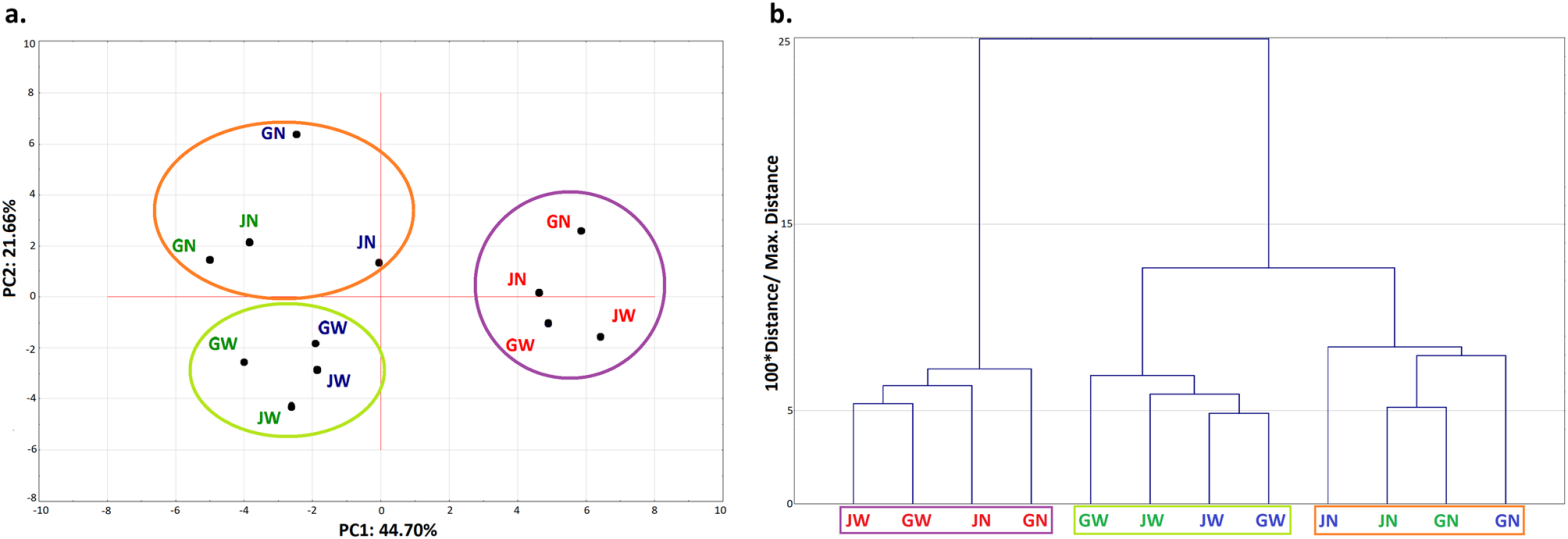
PCA analysis for VOCs and cupping for the roasted coffees. (**a**) Projection of factors on the plane. (**b**) Tree diagram. The font in color blue refers to storage in (– 10 °C), green- 10 °C, and red- 20 °C chambers in time of storage (Statistica, ver. 10, StatSoft).

The performed statistical correlation of VOCs and cupping data (Fig. 4). Figure 4 represents the principal component analysis (PCA) of the chemical and sensorial characteristics of roasted coffee grains after 12 months of storage under different conditions. The presented charts graphically shows the partitioning of the analysed roasted coffees in terms of their postharvest treatment method into three main groups:

- Grains from – 10 °C and 10 °C chambers from Natural treatment (orange ellipse);
- Grains from – 10 °C and 10 °C chambers from Washed treatment (lime ellipse);
- Grains stored in the 20 °C chamber (purple ellipse).

Factors with eigenvalues greater than one accounted for 66.36% of the data variance. The first distinguishing factor (PC1) explained nearly 44.70% of the data variance, and its eigenvalue of 17.8 indicated that it contains information originally explained by nearly 18 variables used to describe the research object. The second distinguishing factor (PC2) explained nearly 21.66% of the data variance, and its eigenvalue of 8.6 indicated that it contains information originally explained by nearly 9 variables used to describe the research object.

PCA allowed us to visually divide the analysed coffee samples into three sectors: **N** CB from – 10 and 10 °C chambers with negative PC1 and positive PC2 values, **W** samples also from − 10 and 10  °C treatment with negative PC1 and negative PC2 values, and CB from 20 °C chamber with positive PC1 values (within this ellipse **W** has negative PC2 values, and **N** has positive PC2 values). Projection unequivocally shows differentiation between **W** and **N** grains, also in the 20 °C chamber tests.

### The influence of storage conditions on changes in the content of free fatty acids (FFAs)

An important indicator of coffee quality is the process of releasing FFAs from the hydrolysis of triglycerides, which is one of the first reactions occurring under the influence of unfavorable post-harvest conditions^31^. As a result of damage to the integrity of the cell membrane^16,32^ in low-quality coffees, a reaction occurs in which the unsaturated fatty acid chains can be easily oxidized to hydroperoxides^33^, which are then converted into several low molecular weight compounds that give the product an unpleasant taste^34^. There are reports that the increase in the number of FFAs can be attributed to the action of lipases or the action of reactive oxygen species^35^. This conclusion may be due to the hydrolytic activity of lipophilic enzymes which are more effective at higher temperatures.

A study by S. Cong et al.^36^ indicated that the higher the storage temperature, the faster the growth of FFAs in coffee beans. Conclusions presented by M.Y. Rendón et al.^37^ also showed a systematic increase in the amount of FFAs over time in terms of 15 months.

**Figure 5:**
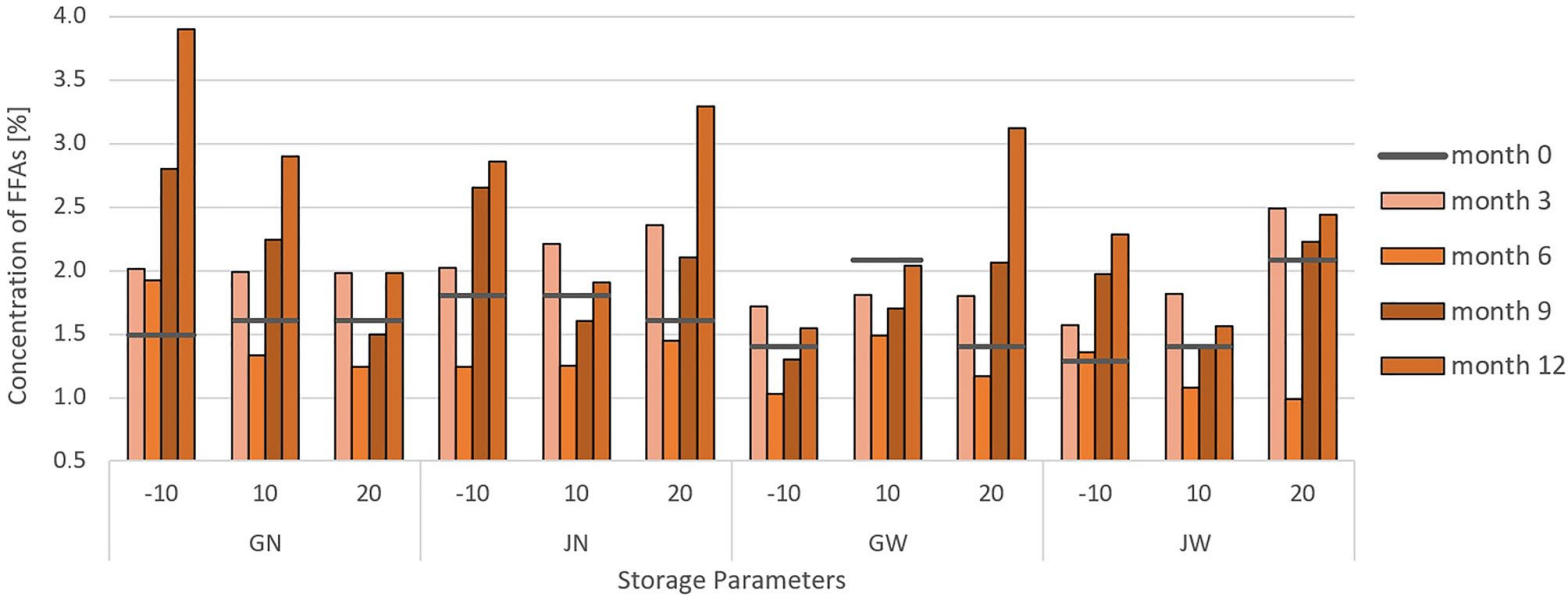
Diagram of average content of FFAs in dry mass analysed in tested green coffee samples on a 12-month basis (with results recorded every 3 months), after appropriate treatment, in packaging.

The experiments performed by our research team confirm the aforementioned conclusions. Changes in the average content of FFAs in the 12-month research period are illustrated in the diagram in Fig. 5.

FFAs analysis is one of the basic parameters of the quality of green coffee beans during storage. The concentration of FFAs from freshly harvested coffee beans is as low as 1 g/kg (concentration of FFAs at level 0.1% of dry mass). The increase in their content is influenced by the temperature and humidity of the environment.

Based on the presented Fig. 5, it was noticed that the concentration of FFAs in the 3rd month (except 10**GW**) exceeds the values from the initial measurement and in the 6th month. After half year of storage, the FFAs value clearly decreases, but for the next half a year, until the 12th month of storage, the values increase.

The content of FFAs were lower in samples of **W** green beans, what can be explained by washing the oily components out of the beans by washing them. That tendency was especially observed in **W** grains from chambers − 10 °C and 10 °C which had lower FFA contents than analogous grains from **N** treatment. The lowest FFAs values were found in grains stored in − 10**GW** and 10**JW**, slightly higher values of 10**GW** and − 10**JW**.

Examining the aspect of the impact of packaging on the concentration of FFAs, it was not noticed that it was a differentiating factor. This is in contrast to the conclusion made in the Flavio et al.^38^ article, where it was stated that jute bags are not recommended for storing specialty coffees. Based on our experiment, we are unable to support this thesis. This may be because in the aforementioned article, the grains in jute bags were stored in different conditions than grains in GrainPro, and in our case, the storage conditions were the same for all packaging.

### The influence of storage conditions on changes in water activity (WA)

**Figure 6:**
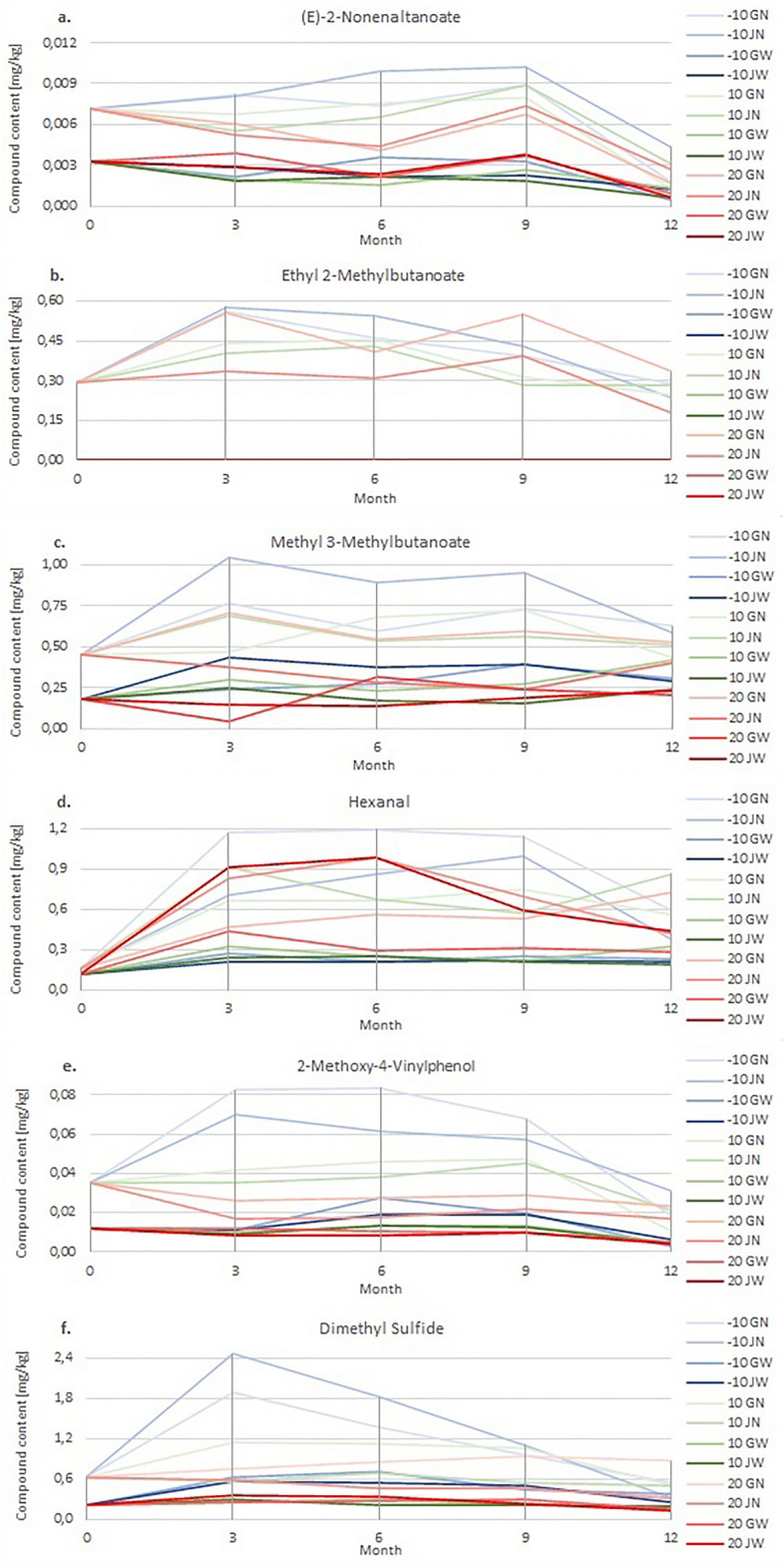
Diagram of average water activity analysed in tested green coffee samples on a 12-month basis (with results recorded every 3 months), after appropriate treatment.

Water activity is a parameter that can be interpreted as the amount of water in a substance that is in an unbound and free form that can take part in chemical reactions^39^. Higher water activity means that more water is available for chemical reactions. The literature indicates that it is best for the tested values for green beans to be above 0.45 which is required to protect the beans during storage to achieve and maintain a sufficiently high quality^39^. Coffee beans can easily absorb moisture from the environment during storage because they are a highly hygroscopic matrix. It has been proven that a water content of 0.85 allows the germination and growth of fungus^40^. Figure 6 shows the graph with the values of 0.45 marked in red.

The presented diagram (Fig. 5) shows that two samples, i.e. 20**JN** and 20**JW** did not meet the expectations of maintaining the water activity level (values < 0.45) after 6 months of storage. Therefore the measurements were not continued for these samples. Among the remaining samples, no WA values above 0.85 were recorded. In the literature, the range of results for Natural processing was between 0.40 to 0.65, and for Washed ones- from 0.35 to 0.70^41^. That was convergent to obtained ranges for our experiment (**N**- 0.40–0.59, **W**- 0.38- 0.61). According to the SCA guidelines, the upper limit of water activity for coffee that can be considered “specialty grade” is 0.70^41^.

The presented graph (Fig. 6) shows the tested samples broken down by the type of packaging, then the storage temperature, and the method of processing. There is a clear difference between the AW values of grains stored in **J** bags and **G**. In natural packages, it was observed that WA decreases with increasing temperature in the storage chamber, and the same for N and W grains. Polymer bags, as intended, hold WA levels much better. Almost all cases (except 20**GW**) record only minor fluctuations of the measured value over the period of 12 months.

### The influence of storage conditions on the quantitative changes of green grain VOCs

Six volatile organic compounds were selected to evaluate the changes taking place in green beans.

(E)-2-Nonenal is the oxidation product of unsaturated fatty acids considered responsible for the characteristic woody aroma that persists in long-stored grains^37^.

Esters represented by methyl 3-Methylbutanoate is formed by the esterification of alcohol and FFA by chemical or enzymatic pathways^42^. Ethyl 2-Methylbutyrate is one of the key fragrances responsible for unpleasant odour notes in long-stored and fermented coffee beans (uncontrolled fermentation)^8^.

Hexanal is known to impart a rancid taste to coffee from the oxidation of polyunsaturated fatty acids, such as linoleic acid, which are abundant in coffee^12,43^. In a study by E. Makri et al.^44^ an exponential increase in hexanal concentration in those stored in an aerobic atmosphere was observed, therefore this compound was suggested as a marker in the coffee storage process.

The roasting pathways of guaiacols (sharp sensory notes) to which 2-Methoxy-4-Vinylphenol belongs include, for example, decarboxylation of phenolic carboxylic acids and thermal degradation of lignin; however, their formation (or concentration) in the coffee aroma also depends on bacterial, fungal, yeast enzymes and the glycosidic reactions taking place in the green bean^45^.

Dimethyl sulfide is known to be associated with an aerobic atmosphere of storage, wet processing^7^, and the presence of microorganisms^46^. Its concentration level can be considered an indicator of poor grain quality^38^.

**Figure 7:**
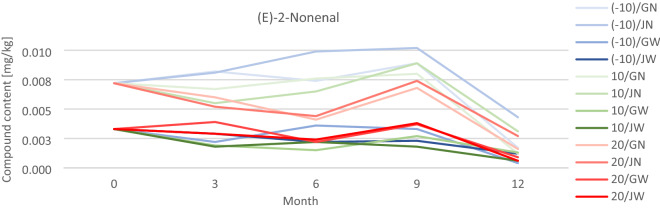
The content of (E)-2-Nonenal in tested green coffee samples on a 12-month basis (with results recorded every 3 months), after appropriate treatment in packaging*.*

Figure 7 shows the changes in the content of (E)-2-Nonenal over time. Graphs for other VOCs are provided in the Supplementary.

The highest content of methyl 3-Methylbutanoate was found in **N** grains up to 6 months of storage.

For green beans, **N** type stored in **J** observed a dependence of the content of Methyl 3-Methylbutanoate on the temperature of their storage. The highest values were obtained for grains from the chamber with a storage temperature of − 10 °C, and the lowest values for grains from the chamber with a temperature of 20 °C.

No Ethyl 2-Methylbutyrate was found in **W** green coffee beans.

Generally, the highest levels of Ethyl 2-Methylbutyrate were found in **N** grains after 3 months of storage.

The highest content of dimethyl sulfide, over 1.5 mg/kg, was found in **N** grains stored for up to 6 months in a chamber at − 10 °C.

For compounds formed as a result of the activity of microorganisms, enzyme activity, and fermentation for samples in **J**, the dependence of their content on temperature was observed. Their highest contents were found in the samples stored in the.

− 10 °C chamber, and the lowest in the 20 °C chamber. Moreover, the content of these compounds generally decreases during storage. Their contents in the **N** samples are higher than in the **W** samples.

Compounds whose presence derives from lipid oxidation, i.e. (E)-2-Nonenal, Hexanal, and 2-Methoxy-4-Vinylphenol, first increased and then decreased during their storage. The obtained results of their content in **W** green coffee beans are characterized by a smaller scatter of results and are lower than the results obtained for **N** beans.

For the lipid oxidation products, no clear dependence of the change in their content on the grain storage temperature was observed.

## Summary

In accordance with the assumptions of the work, it was shown how post-harvest grain processing, packaging material and temperature influenced sensory characteristics, FFAs, WA, VOCs. The results presented in the study clearly show that the storage of grains in a chamber at a temperature of 20 °C does not allow for their freshness and high quality as long as the other two tested temperature chambers. The − 10 °C and 10 °C chambers showed no particularly dissimilar results. Better quality parameters were selected among the results obtained for the **N** grains. No clear differences were observed between **J** and **G** bags. The conclusions received are valuable information for intermediaries in the coffee supply chain, because as stated in the introduction, coffee production is seasonal, and its consumption is all year round, therefore the storage of beans at the right conditions plays a key role among post-collective factors.

## Supplementary Information

Supplementary Information.

## Data Availability

All related data are available in the form of electronic Supplementary materials. All relevant data are included in the supplementary materials of the manuscript. Correspondence and requests for materials should be addressed to J.B.

## Abbreviations

CB: Coffee beans
W: Washed
N: Natural
G: GrainPro bag
J: Jute bag
VOCs: Volatile organic compounds
FFAs: Free fatty acids
WA: Water activity

## References

[1] Nijssen LM (1996). Volatile Compounds in Food: Qualitative and Quantitative Data.

[2] Flamen I (1989). Coffee, cocoa, and tea. Food Rev. Int..

[3] Mayer F, Czerny M, Grosch W (2000). Sensory study of the character impact aroma compounds of a coffee beverage. Eur. Food Res. Technol..

[4] International Coffee Organization - Annual Review 2018/2019 ‘Addressing the Coffee Price Crisis’. https://new.ico.org/show_news.asp?id=730.

[5] Ismail I, Anuar MS, Shamsudin R (2013). Effect on the physicochemical properties of liberica green coffee beans under ambient storage. Int. Food Res. J..

[6] Haile M, Hee Kang W, Toledo Castanheira D (2020). The harvest and post-harvest management practices’ impact on coffee quality. Coffee - Production and Research.

[7] Toledo PRAB, Pezza L, Pezza HR, Toci AT (2016). Relationship between the different aspects related to coffee quality and their volatile compounds: Volatile compounds and coffee quality. Compr. Rev. Food Sci. Food Saf..

[8] Hameed A (2018). Farm to consumer: Factors affecting the organoleptic characteristics of coffee. II: Postharvest processing factors: Farm to consumer. Compr. Rev. Food Sci. Food Saf..

[9] de Melo Pereira GV, Soccol VT, Brar SK, Neto E, Soccol CR (2017). Microbial ecology and starter culture technology in coffee processing. Crit. Rev. Food Sci. Nutr..

[10] Illy E (2002). The complexity of coffee. Sci. Am..

[11] Leino M, Kaitaranta J, Kallio H (1992). Comparison of changes in headspace volatiles of some coffee blends during storage. Food Chem..

[12] Amstalden LC, Leite F, de Menezes HC (2001). Identificação e quantificação de voláteis de café através de cromatografia gasosa de alta resolução/espectrometria de massas empregando um amostrador automático de" headspace". Food Sci. Technol..

[13] Marin K, Požrl T, Zlatić E, Plestenjak A (2008). A new aroma index to determine the aroma quality of roasted and ground coffee during storage. Food Technol. Biotechnol..

[14] Physical chemistry of roasted and ground coffee: Shelf life improvement for flexible packaging | ASIC—Association for science and information on coffee. https://www.asic-cafe.org/conference/19th-international-scientific-colloquium-coffee/physical-chemistry-roasted-and-ground.

[15] Bonnländer B, Cappuccio R, Liverani FS, Winterhalter P (2006). Analysis of enantiomeric linalool ratio in green and roasted coffee. Flavour Fragr. J..

[16] Vila MA, Andueza S, Paz de Peña M, Cid C (2005). Fatty acid evolution during the storage of ground, roasted coffees. J. Am. Oil Chem. Soc..

[17] Bandeira RD, Toci AT, Trugo LC, Farah A (2009). Volatile composition of intrinsic defective coffee beans by GC/MS-headspace. Quím. Nova.

[18] Bröhan M, Huybrighs T, Wouters C, Van der Bruggen B (2009). Influence of storage conditions on aroma compounds in coffee pads using static headspace GC–MS. Food Chem..

[19] Buffo RA, Cardelli-Freire C (2004). Coffee flavour: An overview. Flavour Fragr. J..

[20] Petisca C, Pérez-Palacios T, Farah A, Pinho O, Ferreira IMPLVO (2013). Furans and other volatile compounds in ground roasted and espresso coffee using headspace solid-phase microextraction: Effect of roasting speed. Food Bioprod. Process..

[21] Poyraz İE, Öztürk N, Kiyan HT, Demirci B (2016). Volatile compounds of *Coffea*
*arabica* L. green and roasted beans. Anadolu Univ. J. Sci. Technol. C Life Sci. Biotechnol..

[22] Post Harvest Handling & Storage Solutions | GrainPro. https://www.grainpro.com.

[23] International Coffee Organization - What’s New. https://www.ico.org/.

[24] PR - CUPPING PROTOCOLS V.21NOV2009A.pdf.

[25] Sanz C, Czerny M, Cid C, Schieberle P (2002). Comparison of potent odorants in a filtered coffee brew and in an instant coffee beverage by aroma extract dilution analysis (AEDA). Eur. Food Res. Technol..

[26] Wang X (2022). Review on factors affecting coffee volatiles: From seed to cup. J. Sci. Food Agric..

[27] Bressanello D (2017). Coffee aroma: Chemometric comparison of the chemical information provided by three different samplings combined with GC–MS to describe the sensory properties in cup. Food Chem..

[28] Czerny M, Grosch W (2000). Potent odorants of raw Arabica coffee. Their changes during roasting. J. Agric. Food Chem..

[29] Variyar PS, Ahmad R, Bhat R, Niyas Z, Sharma A (2003). Flavoring components of raw monsooned arabica coffee and their changes during radiation processing. J. Agric. Food Chem..

[30] Scheidig C, Czerny M, Schieberle P (2007). Changes in key odorants of raw coffee beans during storage under defined conditions. J. Agric. Food Chem..

[31] Marques ER, Borém FM, Pereira RGFA, Biaggioni MAM (2008). Eficácia do teste de acidez graxa na avaliação da qualidade do café Arábica (*Coffea*
*arabica* L.) submetido a diferente períodos e temperaturas de secagem. Ciênc. Agrotecnol..

[32] Dussert S (2006). Oxidative stress, phospholipid loss and lipid hydrolysis during drying and storage of intermediate seeds. Physiol. Plant..

[33] Coradi PC, Borém FM, Saath R, Marques ER (2007). Effect of drying and storage conditions on the quality of natural anda washed coffee. Coffee Sci..

[34] Gutkoski LC, El-Dash AA (1999). Efeito do cozimento por extrusão na estabilidade oxidativa de produtos de moagem de aveia. Pesqui. Agropecu. Bras..

[35] Speer K, Kölling-Speer I (2006). The lipid fraction of the coffee bean. Braz. J. Plant Physiol..

[36] Cong S (2020). Characterization of the lipid oxidation process of robusta green coffee beans and shelf life prediction during accelerated storage. Molecules.

[37] Rendón MY, de Jesus Garcia Salva T, Bragagnolo N (2014). Impact of chemical changes on the sensory characteristics of coffee beans during storage. Food Chem..

[38] Borém FM (2019). Sensory analysis and fatty acid profile of specialty coffees stored in different packages. J. Food Sci. Technol..

[39] Aung Moon S, Wongsakul S, Kitazawa H, Saengrayap R (2022). Lipid oxidation changes of Arabica green coffee beans during accelerated storage with different packaging types. Foods.

[40] Pardo E, Ramos A, Sanchis V, Marin S (2005). Modelling of effects of water activity and temperature on germination and growth of ochratoxigenic isolates of on a green coffee-based medium. Int. J. Food Microbiol..

[41] Qian M, Nelson C, Bloomer S (2002). Evaluation of fat-derived aroma compounds in blue cheese by dynamic headspace GC/Olfactometry-MS. J. Am. Oil Chem. Soc..

[42] Toci AT, Neto VJMF, Torres AG, Farah A (2013). Changes in triacylglycerols and free fatty acids composition during storage of roasted coffee. LWT Food Sci. Technol..

[43] Makri E, Tsimogiannis D, Dermesonluoglu EK, Taoukisa PS (2011). Modeling of Greek coffee aroma loss during storage at different temperatures and water activities. Procedia Food Sci..

[44] Flament I (2001). Coffee Flavor Chemistry.

[45] Spadone JC, Takeoka G, Liardon R (1990). Analytical investigation of Rio off-flavor in green coffee. J. Agric. Food Chem..

[46] Toci AT, Farah A (2014). Volatile fingerprint of Brazilian defective coffee seeds: Corroboration of potential marker compounds and identification of new low quality indicators. Food Chem..

