# Supplementary material for: Effect of green and roasted coffee storage conditions on selected characteristic quality parameters
Source: Sci Rep. 2023 Apr 20;13:6447. doi: 10.1038/s41598-023-33609-x (PMC10119118; doi:10.1038/s41598-023-33609-x)
Supplement: Supplementary file 1 — Supplementary Information. [file 41598_2023_33609_MOESM1_ESM.docx]

**Effect of green and roasted coffee storage conditions on selected characteristic quality parameters**

Justyna Błaszkiewicz*^1^, Ewa Nowakowska‑Bogdan^1^, Krzysztof Barabosz^2^, Renata Kulesza^1^, Ewa Dresler^1^, Piotr Woszczyński^1^, Łukasz Biłos^3^, Dominika Barbara Matuszek^3^, Krzysztof Szkutnik^4^

^1^ The Łukasiewicz Research Network-Institute of Heavy Organic Synthesis “Blachownia”, Energetykow 9, 47-225, Kedzierzyn-Kozle, Poland;

^2^ Mechanical Engineering, Opole University of Technology Doctoral School, Prószkowska 76, 45-758 Opole, Poland;

^3^ Faculty of Production Engineering and Logistics, Opole University of Technology, Proszkowska 76, 45-758, Opole, Poland;

^4^ Hard Beans Coffee Roasters Sp. z o.o., Armii Krajowej 35, 45-071 Opole, Poland.

Supplementary Information

[***Figure 1.*** *2*](#_Toc129954160)

[***Figure 2****. 3*](#_Toc129954161)

[***Figure 3****. 4*](#_Toc129954162)

[***Figure 4.****. 5*](#_Toc129954163)

[***Figure 5.*** *5*](#_Toc129954164)

[***Figure 6.*** *…………………………………………………………………………………………………………………………………………….6*](#_Toc129954165)

[***Table 1.*** *7*](#_Toc129954173)

[***Table 2.****. 10*](#_Toc129954174)

[***Table 3.****. 11*](#_Toc129954175)

[***Table 4****. . 11*](#_Toc129954176)

[***Table 5.****. 12*](#_Toc129954177)

[***Table 6.****. 12*](#_Toc129954178)


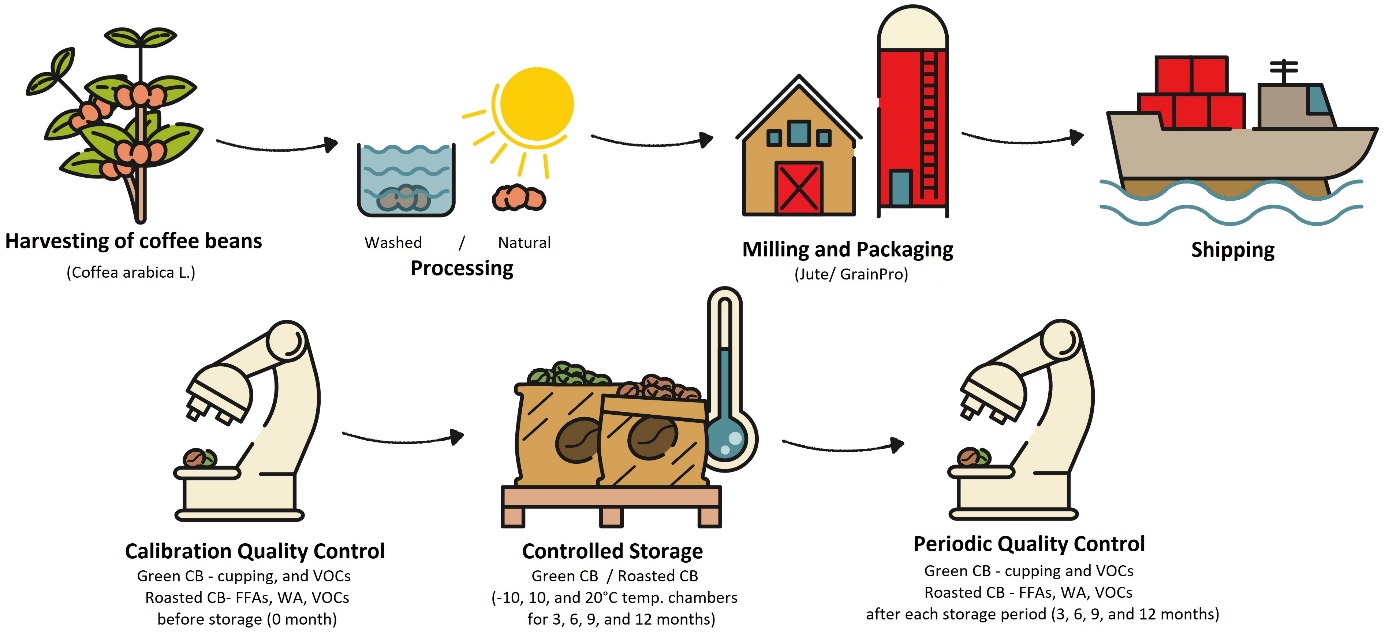


**Figure 1.** Illustration showing the process that the coffee beans have gone through from picking to analysis.


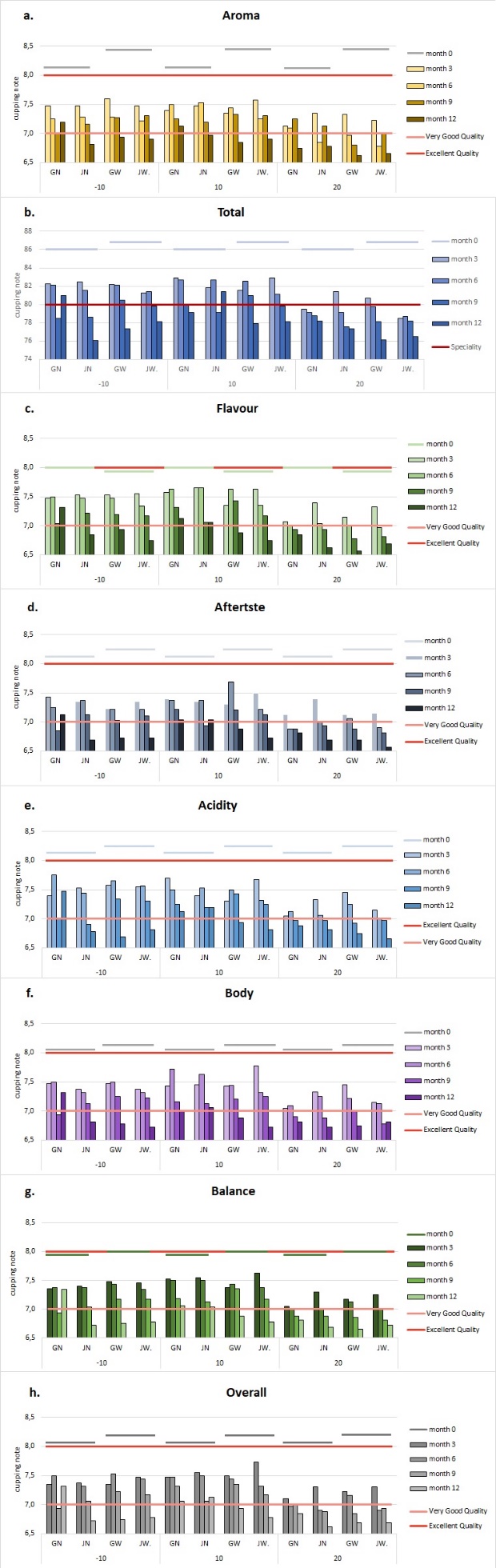


**Figure 2**. Graph of results of the sensory evaluation for 2a. Aroma , 2b. Total note, 2c. Flavour, 2d. Aftertaste, 2e.Acidity, 2f. Body, 1f. Balance, 1h. Overall note in the initial roasted coffee and after storage for 3, 6, 9 and 12 months (**N**- Natural, **W**- Washed treatment) in packaging (**J**- Jute bags, **G**- GrainPro bags).


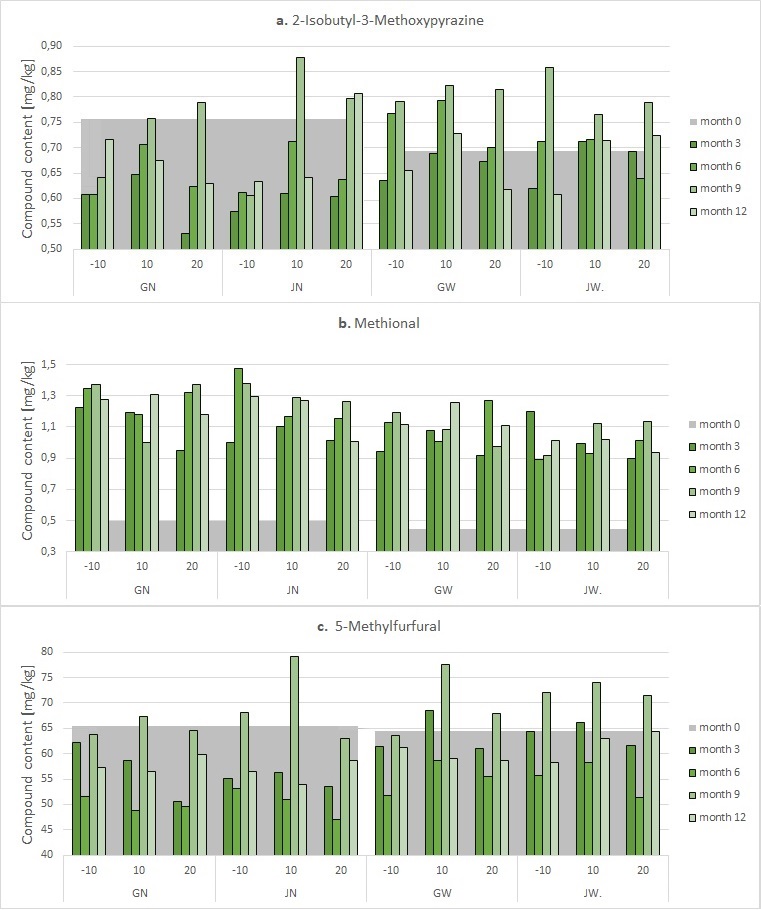


**Figure 3**. Graph of changes of content of compound in tested roasted coffee samples on a 12-month basis (with results recorded every 3 months), after appropriate treatment (**N**- Natural, **W**- Washed) in packaging (**J**- Jute bags, **G**- GrainPro bags). **a**. 2-Isopropyl-3-Methoxypyrazine; **b**. Methional; **c.** 5-Methylfurfural.


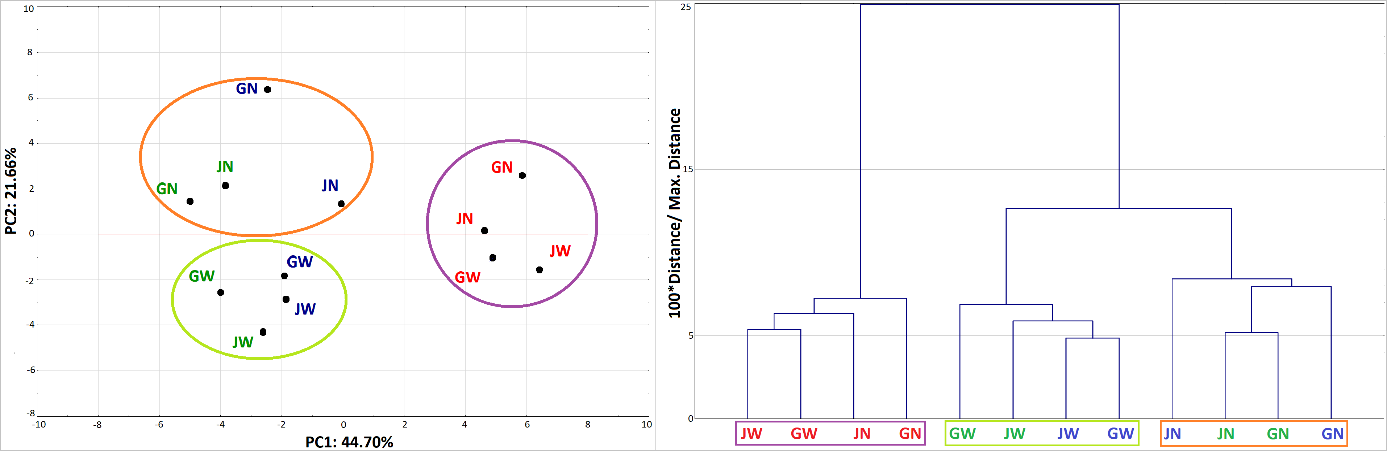


**Figure 4.** PCA analysis for VOCs and cupping for the roasted coffees. **a.** Projection of factors on the plane. **b.** Tree diagram. Font in colour blue refers to storage in (-10⁰C), green- 10⁰C, red- 20⁰C chambers in time of storage (Statistica, ver. 10, StatSoft). (**N**- Natural, **W**- Washed treatment) in packaging (**J**- Jute bags, **G**- GrainPro bags).


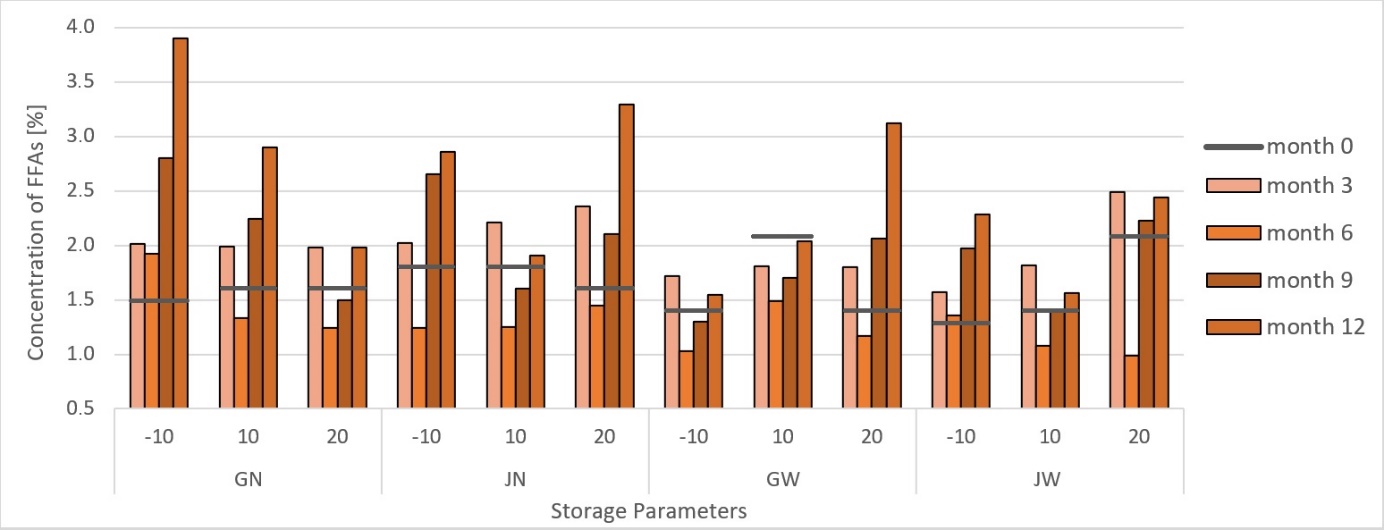


**Figure 5.** Diagram of average content of FFAs in dry mass analysed in tested green coffee samples on a 12-month basis (with results recorded every 3 months), after appropriate treatment (**N**- Natural, **W**- Washed) in packaging (**J**- Jute bags, **G**- GrainPro bags).


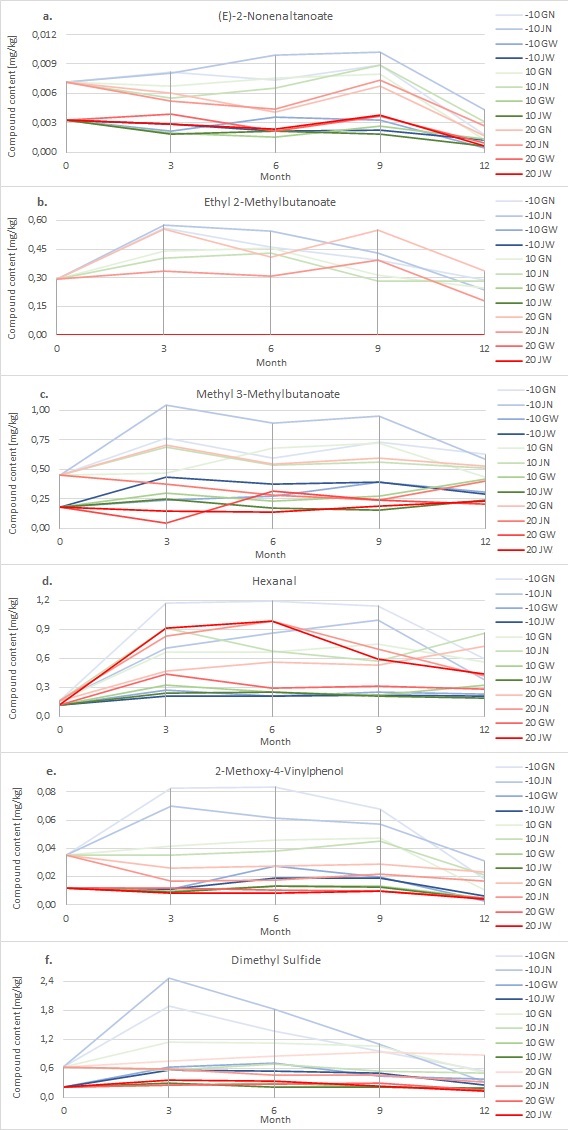


**Figure 6.** Diagram of average water activity analysed in tested green coffee samples on a 12-month basis (with results recorded every 3 months), after appropriate treatment. **a.** (E)-2-Nonenal, **b.** Ethyl 2-Methylbutyrate, **c.** Methyl 3-Methylbutanoate, **d.** Hexanal, **e.** 2-Methoxy-4-Vinylphenol, **f.** Dimethyl Sulfide**.**

**Table 1.** Statistical correlations between individual parameters. Red results: correlation coefficient with an absolute value greater than 0.6 and with a p value < 0.05.

|  |  |  | VOCs | | | Cupping attributes | | | | | | |
| --- | --- | --- | --- | --- | --- | --- | --- | --- | --- | --- | --- | --- |
|  |  |  | Methional | 5-Methylfurfural | 2-Isopropyl-3-Methoxypyrazine | Aroma | Flavour | Aftertaste | Acidity | Body | Balance | Overall |
| 3 months | VOCs | Methional | 1.0000 | 0.2099 | 0.-1280 | 0.3358 | 0.5268 | 0.6452 | 0.3424 | 0.2551 | 0.4457 | 0.4074 |
|  |  |  |  | p= 0.513 | p= 0.692 | p= 0.286 | p= 0.078 | p= 0.023 | p= 0.276 | p= 0.424 | p= 0.146 | p= 0.189 |
|  |  | 5-Methylfurfural | 0.2099 | 1.0000 | 0.8095 | 0.4129 | 0.2703 | 0.2251 | 0.2083 | 0.5788 | 0.4272 | 0.5857 |
|  |  |  | p= 0.513 |  | p= 0.001 | p= 0.182 | p= 0.395 | p= 0.482 | p= 0.516 | p= 0.049 | p= 0.166 | p= 0.045 |
|  |  | 2-Isopropyl-3-Methoxypyrazine | -0.1280 | 0.8095 | 1.0000 | 0.2369 | 0.1889 | 0.1185 | 0.2466 | 0.5374 | 0.3815 | 0.5381 |
|  |  |  | p= 0.692 | p= 0.001 |  | p= 0.459 | p= 0.557 | p= 0.714 | p= 0.440 | p= 0.072 | p= 0.221 | p= 0.071 |
|  | Cupping attributes | Aroma | 0.3358 | 0.4129 | 0.2369 | 1.0000 | 0.8368 | 0.6466 | 0.7572 | 0.8314 | 0.8476 | 0.6894 |
|  |  |  | p= 0.286 | p= 0.182 | p= 0.459 |  | p= 0.001 | p= 0.023 | p= 0.004 | p= 0.001 | p= 0.000 | p= 0.013 |
|  |  | Flavour | 0.5268 | 0.2703 | 0.1889 | 0.8368 | 1.0000 | 0.7881 | 0.7921 | 0.6440 | 0.9550 | 0.8181 |
|  |  |  | p= 0.078 | p= 0.395 | p= 0.557 | p= 0.001 |  | p= 0.002 | p= 0.002 | p= 0.024 | p= 0.000 | p= 0.001 |
|  |  | Aftertaste | 0.6452 | 0.2251 | 0.1185 | 0.6466 | 0.7881 | 1.0000 | 0.7101 | 0.6699 | 0.7546 | 0.7497 |
|  |  |  | p= 0.023 | p= 0.482 | p= 0.714 | p= 0.023 | p= 0.002 |  | p= 0.010 | p= 0.017 | p= 0.005 | p= 0.005 |
|  |  | Acidity | 0.3424 | 0.2083 | 0.2466 | 0.7572 | 0.7921 | 0.7101 | 1.0000 | 0.6599 | .8086 | .6371 |
|  |  |  | p= 0.276 | p= 0.516 | p= 0.440 | p= 0.004 | p= 0.002 | p= 0.010 |  | p= 0.020 | p= 0.001 | p= 0.026 |
|  |  | Body | 0.2551 | 0.5788 | 0.5374 | 0.8314 | 0.6440 | 0.6699 | 0.6599 | 1.0000 | 0.7729 | 0.7888 |
|  |  |  | p= 0.424 | p= 0.049 | p= 0.072 | p= 0.001 | p= 0.024 | p= 0.017 | p= 0.020 |  | p= 0.003 | p= 0.002 |
|  |  | Balance | 0.4457 | 0.4272 | 0.3815 | 0.8476 | 0.9550 | 0.7546 | 0.8086 | 0.7729 | 1.0000 | 0.9173 |
|  |  |  | p= 0.146 | p= 0.166 | p= 0.221 | p= 0.000 | p= 0.000 | p= 0.005 | p= 0.001 | p= 0.003 |  | p= 0.000 |
|  |  | Overall | 0.4074 | 0.5857 | 0.5381 | 0.6894 | 0.8181 | 0.7497 | 0.6371 | 0.7888 | 0.9173 | 1.0000 |
|  |  |  | p= 0.189 | p= 0.045 | p= 0.071 | p= 0.013 | p= 0.001 | p= 0.005 | p= 0.026 | p= 0.002 | p= 0.000 |  |
| 6 months | VOCs | Methional | 1.0000 | -0.4267 | -0.6068 | 0.0150 | -0.0226 | -0.0870 | 0.0682 | 0.0086 | -0.0907 | -0.0701 |
|  |  |  |  | p= 0.167 | p= 0.036 | p= 0.963 | p= 0.944 | p= 0.788 | p= 0.833 | p= 0.979 | p= 0.779 | p= 0.829 |
|  |  | 5-Methylfurfural | -0.4267 | 1.0000 | 0.5349 | 0.2194 | 0.2035 | 0.4553 | 0.2271 | 0.0987 | 0.3219 | 0.3310 |
|  |  |  | p= 0.167 |  | p= 0.073 | p= 0.493 | p= 0.526 | p= 0.137 | p= 0.478 | p= 0.760 | p= 0.308 | p= 0.293 |
|  |  | 2-Isopropyl-3-Methoxypyrazine | -0.6068 | 0.5349 | 1.0000 | 0.4877 | 0.4576 | 0.5677 | 0.3626 | 0.4233 | 0.5386 | 0.5294 |
|  |  |  | p= 0.036 | p= 0.073 |  | p= 0.108 | p= 0.135 | p= 0.054 | p= 0.247 | p= 0.170 | p= 0.071 | p= 0.077 |
|  | Cupping attributes | Aroma | 0.0150 | 0.2194 | 0.4877 | 1.0000 | 0.9408 | 0.8280 | 0.7684 | 0.8270 | 0.9262 | 0.8701 |
|  |  |  | p= 0.963 | p= 0.493 | p= 0.108 |  | p= 0.000 | p= 0.001 | p= 0.004 | p= 0.001 | p= 0.000 | p= 0.000 |
|  |  | Flavour | -0.0226 | 0.2035 | 0.4576 | 0.9408 | 1.0000 | 0.8964 | 0.8354 | 0.8856 | 0.9657 | 0.9086 |
|  |  |  | p= 0.944 | p= 0.526 | p= 0.135 | p= 0.000 |  | p= 0.000 | p= 0.001 | p= 0.000 | p= 0.000 | p= 0.000 |
|  |  | Aftertaste | -0.0870 | 0.4553 | 0.5677 | 0.8280 | 0.8964 | 1.0000 | 0.6862 | 0.7022 | 0.8544 | 0.7797 |
|  |  |  | p= 0.788 | p= 0.137 | p= 0.054 | p= 0.001 | p= 0.000 |  | p= 0.014 | p= 0.011 | p= 0.000 | p= 0.003 |
|  |  | Acidity | 0.0682 | 0.2271 | 0.3626 | 0.7684 | 0.8354 | 0.6862 | 1.0000 | 0.7564 | 0.8645 | 0.9594 |
|  |  |  | p= 0.833 | p= 0.478 | p= 0.247 | p= 0.004 | p= 0.001 | p= 0.014 |  | p= 0.004 | p= 0.000 | p= 0.000 |
|  |  | Body | 0.0086 | -0.0987 | 0.4233 | 0.8270 | 0.8856 | 0.7022 | 0.7564 | 1.0000 | 0.8651 | 0.8269 |
|  |  |  | p= 0.979 | p= 0.760 | p= 0.170 | p= 0.001 | p= 0.000 | p= 0.011 | p= 0.004 |  | p= 0.000 | p= 0.001 |
|  |  | Balance | -0.0907 | 0.3219 | 0.5386 | 0.9262 | 0.9657 | 0.8544 | 0.8645 | 0.8651 | 1.0000 | 0.9602 |
|  |  |  | p= 0.779 | p= 0.308 | p= 0.071 | p= 0.000 | p= 0.000 | p= 0.000 | p= 0.000 | p= 0.000 |  | p= 0.000 |
|  |  | Ollllll | -0.0701 | 0.3310 | 0.5294 | 0.8701 | 0.9086 | 0.7797 | 0.9594 | 0.8269 | 0.9602 | 1.0000 |
|  |  |  | p= 0.829 | p= 0.293 | p= 0.077 | p= 0.000 | p= 0.000 | p= 0.003 | p= 0.000 | p= 0.001 | p= 0.000 |  |
| 9 months | VOCs | Methional | 1.0000 | -0.3081 | -0.5254 | -0.0306 | -0.1491 | -0.3860 | -0.4741 | -0.3675 | -0.3441 | -0.3632 |
|  |  |  |  | p= 0.330 | p= 0.079 | p= 0.925 | p= 0.644 | p= 0.215 | p= 0.119 | p= 0.240 | p= 0.273 | p= 0.246 |
|  |  | 5-Methylfurfural | -0.3081 | 1.0000 | 0.4613 | 0.2741 | 0.3199 | 0.3211 | 0.4666 | 0.3909 | 0.5272 | 0.3906 |
|  |  |  | p= 0.330 |  | p= 0.131 | p= 0.389 | p= 0.311 | p= 0.309 | p= 0.126 | p= 0.209 | p= 0.078 | p= 0.209 |
|  |  | 2-Isopropyl-3-Methoxypyrazine | -0.5254 | 0.4613 | 1.0000 | 0.2005 | 0.1059 | 0.0324 | 0.4533 | 0.1386 | 0.2094 | 0.1488 |
|  |  |  | p= 0.079 | p= 0.131 |  | p= 0.532 | p= 0.743 | p= 0.920 | p= 0.139 | p= 0.667 | p= 0.514 | p= 0.644 |
|  | Cupping attributes | Aroma | -0.0306 | 0.2741 | 0.2005 | 1.0000 | 0.7753 | 0.6967 | 0.7340 | 0.6436 | 0.7622 | 0.8072 |
|  |  |  | p= 0.925 | p= 0.389 | p= 0.532 |  | p= 0.003 | p= 0.012 | p= 0.007 | p= 0.024 | p= 0.004 | p= 0.002 |
|  |  | Flavour | -0.1491 | 0.3199 | 0.1059 | 0.7753 | 1.0000 | 0.9095 | 0.7589 | 0.7910 | 0.9248 | 0.9194 |
|  |  |  | p= 0.644 | p= 0.311 | p= 0.743 | p= 0.003 |  | p= 0.000 | p= 0.004 | p= 0.002 | p= 0.000 | p= 0.000 |
|  |  | Aftertaste | -0.3860 | 0.3211 | -0.0324 | 0.6967 | 0.9095 | 1.0000 | 0.6720 | 0.8058 | 0.8538 | 0.8646 |
|  |  |  | p= 0.215 | p= 0.309 | p= 0.920 | p= 0.012 | p= 0.000 |  | p= 0.017 | p= 0.002 | p= 0.000 | p= 0.000 |
|  |  | Acidity | -0.4741 | 0.4666 | 0.4533 | 0.7340 | 0.7589 | 0.6720 | 1.0000 | 0.7888 | 0.9176 | 0.8859 |
|  |  |  | p= 0.119 | p= 0.126 | p= 0.139 | p= 0.007 | p= 0.004 | p= 0.017 |  | p= 0.002 | p= 0.000 | p= 0.000 |
|  |  | Body | -0.3675 | 0.3909 | 0.1386 | 0.6436 | 0.7910 | 0.8058 | 0.7888 | 1.0000 | 0.8964 | 0.8034 |
|  |  |  | p= 0.240 | p= 0.209 | p= 0.667 | p= 0.024 | p= 0.002 | p= 0.002 | p= 0.002 |  | p= 0.000 | p= 0.002 |
|  |  | Balance | -0.3441 | 0.5272 | 0.2094 | 0.7622 | 0.9248 | 0.8538 | 0.9176 | 0.8964 | 1.0000 | 0.9354 |
|  |  |  | p= 0.273 | p= 0.078 | p= 0.514 | p= 0.004 | p= 0.000 | p= 0.000 | p= 0.000 | p= 0.000 |  | p= 0.000 |
|  |  | Overall | -0.3632 | 0.3906 | 0.1488 | 0.8072 | 0.9194 | 0.8646 | 0.8859 | 0.8034 | 0.9354 | 1.0000 |
|  |  |  | p= 0.246 | p= 0.209 | p= 0.644 | p= 0.002 | p= 0.000 | p= 0.000 | p= 0.000 | p= 0.002 | p= 0.000 |  |
| 12 months | VOCs | Methional | 1.0000 | -0.7402 | -0.2655 | 0.5396 | 0.7276 | 0.7596 | 0.6580 | 0.6590 | 0.6161 | 0.6861 |
|  |  |  |  | p= 0.006 | p= 0.404 | p= 0.070 | p= 0.007 | p= 0.004 | p= 0.020 | p= 0.020 | p= 0.033 | p= 0.014 |
|  |  | 5-Methylfurfural | -0.7402 | 1.0000 | 0.2993 | -0.4423 | -0.4833 | -0.6563 | -0.6178 | -0.5056 | -0.4722 | -0.5313 |
|  |  |  | p= 0.006 |  | p= 0.345 | p= 0.150 | p= 0.111 | p= 0.020 | p= 0.032 | p= 0.094 | p= 0.121 | p= 0.075 |
|  |  | 2-Isopropyl-3-Methoxypyrazine | -0.2655 | 0.2993 | 1.0000 | 0.0501 | -0.0477 | -0.0216 | 0.0871 | 0.0666 | 0.0770 | -0.0309 |
|  |  |  | p= 0.404 | p= 0.345 |  | p= 0.877 | p= 0.883 | p= 0.947 | p= 0.788 | p= 0.837 | p= 0.812 | p= 0.924 |
|  | Cupping attributes | Aroma | 0.5396 | -0.4423 | 0.0501 | 1.0000 | 0.8934 | 0.8187 | 0.7927 | 0.7268 | 0.8543 | 0.8138 |
|  |  |  | p= 0.070 | p= 0.150 | p= 0.877 |  | p= 0.000 | p= 0.001 | p= 0.002 | p= 0.007 | p= 0.000 | p= 0.001 |
|  |  | Flavour | 0.7276 | -0.4833 | -0.0477 | 0.8934 | 1.0000 | 0.8903 | 0.8573 | 0.8984 | 0.9291 | 0.9216 |
|  |  |  | p= 0.007 | p= 0.111 | p= 0.883 | p= 0.000 |  | p= 0.000 | p= 0.000 | p= 0.000 | p= 0.000 | p= 0.000 |
|  |  | Aftertaste | 0.7596 | -0.6563 | -0.0216 | 0.8187 | 0.8903 | 1.0000 | 0.9565 | 0.8817 | 0.9349 | 0.9630 |
|  |  |  | p= 0.004 | p= 0.020 | p= 0.947 | p= 0.001 | p= 0.000 |  | p= 0.000 | p= 0.000 | p= 0.000 | p= 0.000 |
|  |  | Acidity | 0.6580 | -0.6178 | 0.0871 | 0.7927 | 0.8573 | 0.9565 | 1.0000 | 0.9338 | 0.9666 | 0.9593 |
|  |  |  | p= 0.020 | p= 0.032 | p= 0.788 | p= 0.002 | p= 0.000 | p= 0.000 |  | p= 0.000 | p= 0.000 | p= 0.000 |
|  |  | Body | 0.6590 | -0.5056 | 0.0666 | 0.7268 | 0.8984 | 0.8817 | 0.9338 | 1.0000 | 0.9559 | 0.9421 |
|  |  |  | p= 0.020 | p= 0.094 | p= 0.837 | p= 0.007 | p= 0.000 | p= 0.000 | p= 0.000 |  | p= 0.000 | p= 0.000 |
|  |  | Balance | 0.6161 | -0.4722 | 0.0770 | 0.8543 | 0.9291 | 0.9349 | 0.9666 | 0.9559 | 1.0000 | 0.9802 |
|  |  |  | p= 0.033 | p= 0.121 | p= 0.812 | p= 0.000 | p= 0.000 | p= 0.000 | p= 0.000 | p= 0.000 |  | p= 0.000 |
|  |  | Overall | 0.6861 | -0.5313 | -0.0309 | 0.8138 | 0.9216 | .9630 | 0.9593 | 0.9421 | 0.9802 | 1.0000 |
|  |  |  | p=.014 | p=.075 | p=.924 | p=.001 | p=.000 | p=.000 | p=.000 | p=.000 | p=.000 |  |

**Table 2.** Cupping notes for individual grain treatments every 3 months.

|  |  | **Aroma** | | | | | **Flavour** | | | | | **Aftertaste** | | | | | **Acidity** | | | | |
| --- | --- | --- | --- | --- | --- | --- | --- | --- | --- | --- | --- | --- | --- | --- | --- | --- | --- | --- | --- | --- | --- |
|  |  | month | | | | | month | | | | | month | | | | | month | | | | |
| Sample Code | | 0 | 3 | 6 | 9 | 12 | 0 | 3 | 6 | 9 | 12 | 0 | 3 | 6 | 9 | 12 | 0 | 3 | 6 | 9 | 12 |
| -10 | GN | 8.125 | 7.475 | 7.250 | 7.000 | 7.188 | 8.000 | 7.475 | 7.500 | 7.031 | 7.313 | 7.810 | 7.425 | 7.250 | 6.844 | 7.125 | 8.130 | 7.400 | 7.750 | 7.000 | 7.469 |
|  | JN | 8.125 | 7.475 | 7.281 | 7.156 | 6.813 | 8.000 | 7.525 | 7.469 | 7.219 | 6.844 | 7.810 | 7.350 | 7.375 | 7.125 | 6.688 | 8.130 | 7.525 | 7.438 | 6.906 | 6.781 |
|  | GW | 8.438 | 7.600 | 7.281 | 7.275 | 6.938 | 7.938 | 7.525 | 7.469 | 7.188 | 6.938 | 7.750 | 7.225 | 7.219 | 7.025 | 6.719 | 8.250 | 7.575 | 7.656 | 7.344 | 6.688 |
|  | JW | 8.438 | 7.475 | 7.219 | 7.300 | 6.906 | 7.938 | 7.550 | 7.344 | 7.175 | 6.750 | 7.750 | 7.350 | 7.219 | 7.100 | 6.719 | 8.250 | 7.550 | 7.563 | 7.300 | 6.813 |
| 10 | GN | 8.125 | 7.400 | 7.500 | 7.250 | 7.125 | 8.000 | 7.575 | 7.625 | 7.313 | 7.125 | 7.810 | 7.400 | 7.375 | 7.219 | 7.031 | 8.130 | 7.700 | 7.500 | 7.250 | 7.125 |
|  | JN | 8.125 | 7.475 | 7.531 | 7.188 | 6.969 | 8.000 | 7.650 | 7.656 | 7.063 | 7.063 | 7.810 | 7.350 | 7.375 | 6.938 | 7.031 | 8.130 | 7.400 | 7.531 | 7.188 | 7.188 |
|  | GW | 8.438 | 7.350 | 7.438 | 7.325 | 6.844 | 7.938 | 7.350 | 7.625 | 7.425 | 6.875 | 7.750 | 7.300 | 7.688 | 7.200 | 6.875 | 8.250 | 7.300 | 7.500 | 7.425 | 6.938 |
|  | JW | 8.438 | 7.575 | 7.250 | 7.300 | 6.906 | 7.938 | 7.625 | 7.354 | 7.175 | 6.750 | 7.750 | 7.500 | 7.219 | 7.125 | 6.719 | 8.250 | 7.675 | 7.313 | 7.250 | 6.813 |
| 20 | GN | 8.125 | 7.125 | 7.094 | 7.250 | 6.750 | 8.000 | 7.075 | 7.000 | 6.938 | 6.844 | 7.810 | 7.125 | 6.875 | 6.875 | 6.813 | 8.130 | 7.050 | 7.125 | 6.969 | 6.875 |
|  | JN | 8.125 | 7.350 | 6.844 | 7.125 | 6.781 | 8.000 | 7.400 | 7.031 | 6.938 | 6.625 | 7.810 | 7.400 | 7.000 | 6.938 | 6.688 | 8.130 | 7.325 | 7.063 | 6.969 | 6.813 |
|  | GW | 8.438 | 7.325 | 6.969 | 6.800 | 6.625 | 7.938 | 7.150 | 7.000 | 6.775 | 6.563 | 7.750 | 7.125 | 7.063 | 6.875 | 6.688 | 8.250 | 7.450 | 7.250 | 6.925 | 6.750 |
|  | JW | 8.438 | 7.225 | 6.781 | 7.000 | 6.656 | 7.938 | 7.325 | 6.969 | 6.813 | 6.688 | 7.750 | 7.150 | 6.906 | 6.813 | 6.563 | 8.250 | 7.150 | 7.000 | 6.969 | 6.656 |
|  |  | **Body** | | | | | **Balance** | | | | | **Overall** | | | | | **Total** | | | | |
|  |  | month | | | | | month | | | | | month | | | | | month | | | | |
| Sample Code | | 0 | 3 | 6 | 9 | 12 | 0 | 3 | 6 | 9 | 12 | 0 | 3 | 6 | 9 | 12 | 0 | 3 | 6 | 9 | 12 |
| -10 | GN | 8.060 | 7.475 | 7.500 | 6.938 | 7.313 | 7.938 | 7.350 | 7.375 | 6.938 | 7.344 | 8.063 | 7.350 | 7.500 | 6.938 | 7.313 | 86.0 | 82.3 | 82.1 | 78.5 | 81.0 |
|  | JN | 8.060 | 7.375 | 7.313 | 7.125 | 6.813 | 7.938 | 7.400 | 7.375 | 7.031 | 6.719 | 8.063 | 7.375 | 7.313 | 7.063 | 6.719 | 86.0 | 82.5 | 81.6 | 78.6 | 76.1 |
|  | GW | 8.130 | 7.475 | 7.500 | 7.250 | 6.781 | 8.000 | 7.475 | 7.438 | 7.169 | 6.750 | 8.188 | 7.350 | 7.531 | 7.225 | 6.750 | 86.8 | 82.2 | 82.1 | 80.5 | 77.3 |
|  | JW | 8.130 | 7.375 | 7.313 | 7.225 | 6.719 | 8.000 | 7.450 | 7.344 | 7.175 | 6.781 | 8.188 | 7.475 | 7.438 | 7.175 | 6.781 | 86.8 | 81.3 | 81.4 | 79.9 | 78.1 |
| 10 | GN | 8.060 | 7.425 | 7.719 | 7.156 | 7.000 | 7.938 | 7.525 | 7.500 | 7.188 | 7.063 | 8.063 | 7.475 | 7.469 | 7.313 | 7.063 | 86.0 | 82.9 | 82.7 | 80.1 | 79.1 |
|  | JN | 8.060 | 7.450 | 7.625 | 7.125 | 7.063 | 7.938 | 7.550 | 7.500 | 7.125 | 7.031 | 8.063 | 7.550 | 7.500 | 7.063 | 7.125 | 86.0 | 81.9 | 82.7 | 79.1 | 81.4 |
|  | GW | 8.130 | 7.425 | 7.438 | 7.200 | 6.875 | 8.000 | 7.375 | 7.438 | 7.350 | 6.875 | 8.188 | 7.500 | 7.438 | 7.350 | 6.938 | 86.8 | 81.6 | 82.6 | 81.0 | 77.9 |
|  | JW | 8.130 | 7.775 | 7.313 | 7.250 | 6.719 | 8.000 | 7.625 | 7.375 | 7.175 | 6.781 | 8.188 | 7.725 | 7.313 | 7.175 | 6.781 | 86.8 | 82.9 | 81.1 | 79.9 | 78.1 |
| 20 | GN | 8.060 | 7.050 | 7.094 | 6.906 | 6.813 | 7.938 | 7.050 | 7.000 | 6.875 | 6.813 | 8.063 | 7.100 | 6.969 | 7.000 | 6.844 | 86.0 | 79.5 | 79.2 | 78.8 | 78.2 |
|  | JN | 8.060 | 7.325 | 7.250 | 6.875 | 6.719 | 7.938 | 7.300 | 7.000 | 6.875 | 6.688 | 8.063 | 7.300 | 6.906 | 6.875 | 6.625 | 86.0 | 81.4 | 79.1 | 77.6 | 77.3 |
|  | GW | 8.130 | 7.450 | 7.219 | 7.000 | 6.750 | 8.000 | 7.175 | 7.125 | 6.850 | 6.656 | 8.188 | 7.225 | 7.156 | 6.850 | 6.688 | 86.8 | 80.7 | 79.8 | 78.2 | 76.1 |
|  | JW | 8.130 | 7.150 | 7.125 | 6.781 | 6.813 | 8.000 | 7.250 | 7.000 | 6.813 | 6.719 | 8.188 | 7.300 | 6.906 | 6.938 | 6.688 | 86.8 | 78.5 | 78.7 | 78.2 | 76.5 |

**Table 3.** Analyzed VOC from green beans values over 12 months (on a 3-monthly basis).

|  |  | 1. **Methional** **[mg/kg]** | | | | | 1. **2-Isopropyl-3-Methoxypyrazine [mg/kg]** | | | | | 1. **5-Methylfurfural [mg/kg]** | | | | |
| --- | --- | --- | --- | --- | --- | --- | --- | --- | --- | --- | --- | --- | --- | --- | --- | --- |
|  |  | month | | | | | month | | | | | month | | | | |
| Sample Code | | 0 | 3 | 6 | 9 | 12 | 0 | 3 | 6 | 9 | 12 | 0 | 3 | 6 | 9 | 12 |
| GN | -10 | 0.50 | 1.23 | 1.35 | 1.38 | 1.28 | 0.76 | 0.61 | 0.61 | 0.64 | 0.72 | 65.40 | 62.20 | 51.50 | 63.85 | 57.25 |
|  | 10 | 0.50 | 1.19 | 1.18 | 1.00 | 1.31 | 0.76 | 0.65 | 0.71 | 0.76 | 0.68 | 65.40 | 58.60 | 48.82 | 67.33 | 56.57 |
|  | 20 | 0.50 | 0.95 | 1.32 | 1.37 | 1.18 | 0.76 | 0.53 | 0.62 | 0.79 | 0.63 | 65.40 | 50.57 | 49.59 | 64.55 | 59.88 |
| JN | -10 | 0.50 | 1.00 | 1.47 | 1.38 | 1.30 | 0.76 | 0.57 | 0.61 | 0.61 | 0.63 | 65.40 | 55.07 | 53.20 | 68.20 | 56.50 |
|  | 10 | 0.50 | 1.11 | 1.17 | 1.29 | 1.27 | 0.76 | 0.61 | 0.71 | 0.88 | 0.64 | 65.40 | 56.30 | 51.00 | 79.10 | 53.86 |
|  | 20 | 0.50 | 1.02 | 1.15 | 1.27 | 1.01 | 0.76 | 0.60 | 0.64 | 0.80 | 0.81 | 65.40 | 53.50 | 47.03 | 62.90 | 58.63 |
| GW | -10 | 0.44 | 0.94 | 1.13 | 1.19 | 1.12 | 0.69 | 0.64 | 0.77 | 0.79 | 0.66 | 64.50 | 61.36 | 51.81 | 63.50 | 61.19 |
|  | 10 | 0.44 | 1.08 | 1.01 | 1.09 | 1.26 | 0.69 | 0.69 | 0.79 | 0.82 | 0.73 | 64.50 | 68.54 | 58.63 | 77.59 | 59.04 |
|  | 20 | 0.44 | 0.92 | 1.27 | 0.98 | 1.11 | 0.69 | 0.67 | 0.70 | 0.81 | 0.62 | 64.50 | 61.02 | 55.59 | 68.00 | 58.63 |
| JW | -10 | 0.44 | 1.20 | 0.89 | 0.92 | 1.02 | 0.69 | 0.62 | 0.71 | 0.86 | 0.61 | 64.50 | 64.30 | 55.62 | 72.14 | 58.22 |
|  | 10 | 0.44 | 0.99 | 0.93 | 1.12 | 1.02 | 0.69 | 0.71 | 0.72 | 0.77 | 0.71 | 64.50 | 66.20 | 58.20 | 74.10 | 63.09 |
|  | 20 | 0.44 | 0.90 | 1.02 | 1.14 | 0.94 | 0.69 | 0.69 | 0.64 | 0.79 | 0.72 | 64.50 | 61.59 | 51.45 | 71.51 | 64.37 |

**Table 4**. Analyzed FFAs over 12 months (on a 3-monthly basis).

|  |  | **Free Fatty Acids [%]** | | | | |
| --- | --- | --- | --- | --- | --- | --- |
|  |  | month | | | | |
|  |  | 0 | 3 | 6 | 9 | 12 |
| GN | -10 | 1.5 | 2.0 | 1.9 | 2.8 | 3.9 |
|  | 10 | 1.6 | 2.0 | 1.3 | 2.2 | 2.9 |
|  | 20 | 1.6 | 2.0 | 1.2 | 1.5 | 2.0 |
| JN | -10 | 1.8 | 2.0 | 1.2 | 2.7 | 2.9 |
|  | 10 | 1.8 | 2.2 | 1.3 | 1.6 | 1.9 |
|  | 20 | 1.6 | 2.4 | 1.5 | 2.1 | 3.3 |
| GW | -10 | 1.4 | 1.7 | 1.0 | 1.3 | 1.6 |
|  | 10 | 2.1 | 1.8 | 1.5 | 1.7 | 2.0 |
|  | 20 | 1.4 | 1.8 | 1.2 | 2.1 | 3.1 |
| JW | -10 | 1.3 | 1.6 | 1.4 | 2.0 | 2.3 |
|  | 10 | 1.4 | 1.8 | 1.1 | 1.4 | 1.6 |
|  | 20 | 2.1 | 2.5 | 1.0 | 2.2 | 2.4 |

**Table 5.** Analyzed WA values over 12 months (every 3 months).

|  |  | **Water Activity** | | | | |
| --- | --- | --- | --- | --- | --- | --- |
|  |  | month | | | | |
|  |  | 0 | 3 | 6 | 9 | 12 |
| -10 | GN | 0.529 | 0.52 | 0.507 | 0.528 | 0.541 |
|  | JN | 0.475 | 0.588 | 0.569 | 0.578 | 0.575 |
|  | GW | 0.574 | 0.573 | 0.552 | 0.559 | 0.576 |
|  | JW | 0.555 | 0.61 | 0.581 | 0.608 | 0.595 |
| 10 | GN | 0.526 | 0.519 | 0.506 | 0.522 | 0.516 |
|  | JN | 0.452 | 0.47 | 0.475 | 0.499 | 0.495 |
|  | GW | 0.567 | 0.561 | 0.543 | 0.531 | 0.57 |
|  | JW | 0.483 | 0.527 | 0.507 | 0.52 | 0.536 |
| 20 | GN | 0.52 | 0.517 | 0.488 | 0.493 | 0.52 |
|  | JN | 0.421 | 0.401 | 0.38 | - | - |
|  | GW | 0.566 | 0.568 | 0.538 | 0.504 | 0.454 |
|  | JW | 0.403 | 0.392 | 0.381 | - | - |

**Table 6.** Analyzed VOC from roasted beans values over 12 months (on a 3-monthly basis).

|  |  | **a. Methyl 3-Methylbutanoate** | | | | | **b. Ethyl 2-Methylbutanoate** | | | | | **c. (E)-2-nonenal** | | | | |
| --- | --- | --- | --- | --- | --- | --- | --- | --- | --- | --- | --- | --- | --- | --- | --- | --- |
|  |  | **month** | | | | | **month** | | | | | **month** | | | | |
| Sample Code | | 0 | 3 | 6 | 9 | 12 | 0 | 3 | 6 | 9 | 12 | 0 | 3 | 6 | 9 | 12 |
| -10 | **GN** | 0.4560 | 0.7657 | 0.5973 | 0.7280 | 0.6288 | 0.2927 | 0.5610 | 0.4601 | 0.3926 | 0.2868 | 0.0072 | 0.0082 | 0.0074 | 0.0089 | 0.0017 |
|  | **JN** | 0.4560 | 1.0440 | 0.8921 | 0.9490 | 0.5882 | 0.2927 | 0.5780 | 0.5438 | 0.4310 | 0.2373 | 0.0072 | 0.0081 | 0.0099 | 0.0102 | 0.0043 |
|  | **GW** | 0.1823 | 0.2359 | 0.2704 | 0.3922 | 0.3107 | 0.0000 | 0.0000 | 0.0000 | 0.0000 | 0.0000 | 0.0033 | 0.0022 | 0.0036 | 0.0033 | 0.0004 |
|  | **JW** | 0.1823 | 0.4340 | 0.3789 | 0.3886 | 0.2937 | 0.0000 | 0.0000 | 0.0000 | 0.0000 | 0.0000 | 0.0033 | 0.0029 | 0.0022 | 0.0023 | 0.0012 |
| 10 | **GN** | 0.4560 | 0.4670 | 0.6781 | 0.7270 | 0.4345 | 0.2927 | 0.4406 | 0.4564 | 0.3141 | 0.2458 | 0.0072 | 0.0067 | 0.0076 | 0.0080 | 0.0012 |
|  | **JN** | 0.4560 | 0.6930 | 0.5380 | 0.5630 | 0.5128 | 0.2927 | 0.4048 | 0.4293 | 0.2830 | 0.2804 | 0.0072 | 0.0055 | 0.0065 | 0.0089 | 0.0031 |
|  | **GW** | 0.1823 | 0.2962 | 0.2290 | 0.2780 | 0.4196 | 0.0000 | 0.0000 | 0.0000 | 0.0000 | 0.0000 | 0.0033 | 0.0019 | 0.0015 | 0.0027 | 0.0013 |
|  | **JW** | 0.1823 | 0.2513 | 0.1707 | 0.1536 | 0.2378 | 0.0000 | 0.0000 | 0.0000 | 0.0000 | 0.0000 | 0.0033 | 0.0018 | 0.0022 | 0.0018 | 0.0006 |
| 20 | **GN** | 0.4560 | 0.7100 | 0.5419 | 0.6000 | 0.5307 | 0.2927 | 0.5550 | 0.4065 | 0.5520 | 0.3348 | 0.0072 | 0.0060 | 0.0041 | 0.0068 | 0.0016 |
|  | **JN** | 0.4560 | 0.3735 | 0.2810 | 0.2395 | 0.4020 | 0.2927 | 0.3365 | 0.3089 | 0.3905 | 0.1781 | 0.0072 | 0.0052 | 0.0044 | 0.0074 | 0.0027 |
|  | **GW** | 0.1823 | 0.0450 | 0.3157 | 0.2370 | 0.2051 | 0.0000 | 0.0000 | 0.0000 | 0.0000 | 0.0000 | 0.0033 | 0.0039 | 0.0022 | 0.0037 | 0.0009 |
|  | **JW** | 0.1823 | 0.1483 | 0.1384 | 0.1887 | 0.2350 | 0.0000 | 0.0000 | 0.0000 | 0.0000 | 0.0000 | 0.0033 | 0.0029 | 0.0024 | 0.0038 | 0.0006 |

|  |  | **d. Hexanal** | | | | | **e. 2-Methoxy-4-Vinylphenol** | | | | | **f. Dimethyl sulfide** | | | | |
| --- | --- | --- | --- | --- | --- | --- | --- | --- | --- | --- | --- | --- | --- | --- | --- | --- |
|  |  | **month** | | | | | **month** | | | | | **month** | | | | |
| Sample Code | | 0 | 3 | 6 | 9 | 12 | 0 | 3 | 6 | 9 | 12 | 0 | 3 | 6 | 9 | 12 |
| -10 | **GN** | 0.1586 | 1.1690 | 1.1926 | 1.1400 | 0.5877 | 0.0356 | 0.0824 | 0.0836 | 0.0677 | 0.0180 | 0.6230 | 1.8860 | 1.3690 | 0.9680 | 0.5416 |
|  | **JN** | 0.1586 | 0.7010 | 0.8645 | 0.9900 | 0.3757 | 0.0356 | 0.0699 | 0.0613 | 0.0575 | 0.0308 | 0.6230 | 2.4640 | 1.8280 | 1.1160 | 0.3295 |
|  | **GW** | 0.1160 | 0.2749 | 0.2136 | 0.2534 | 0.2300 | 0.0118 | 0.0109 | 0.0273 | 0.0194 | 0.0027 | 0.2269 | 0.6400 | 0.7166 | 0.4400 | 0.3868 |
|  | **JW.** | 0.1160 | 0.2080 | 0.2132 | 0.2175 | 0.2078 | 0.0118 | 0.0112 | 0.0193 | 0.0186 | 0.0061 | 0.2269 | 0.5620 | 0.5480 | 0.5080 | 0.2543 |
| 10 | **GN** | 0.1586 | 0.6621 | 0.6599 | 0.7430 | 0.5575 | 0.0356 | 0.0419 | 0.0456 | 0.0474 | 0.0101 | 0.6230 | 1.1570 | 1.1190 | 1.0663 | 0.5033 |
|  | **JN** | 0.1586 | 0.9120 | 0.6750 | 0.5660 | 0.8607 | 0.0356 | 0.0351 | 0.0384 | 0.0450 | 0.0208 | 0.6230 | 0.5630 | 0.6960 | 0.5400 | 0.5093 |
|  | **GW** | 0.1160 | 0.3243 | 0.2555 | 0.2202 | 0.3199 | 0.0118 | 0.0094 | 0.0136 | 0.0134 | 0.0050 | 0.2269 | 0.2599 | 0.3050 | 0.2149 | 0.2002 |
|  | **JW.** | 0.1160 | 0.2378 | 0.2548 | 0.2094 | 0.1889 | 0.0118 | 0.0091 | 0.0131 | 0.0126 | 0.0044 | 0.2269 | 0.3100 | 0.2180 | 0.2261 | 0.2039 |
| 20 | **GN** | 0.1586 | 0.4710 | 0.5564 | 0.5326 | 0.7288 | 0.0356 | 0.0261 | 0.0273 | 0.0290 | 0.0230 | 0.6230 | 0.7620 | 0.8520 | 0.9420 | 0.8750 |
|  | **JN** | 0.1586 | 0.8300 | 0.9796 | 0.6927 | 0.4177 | 0.0356 | 0.0169 | 0.0176 | 0.0217 | 0.0167 | 0.6230 | 0.5930 | 0.4745 | 0.4627 | 0.3312 |
|  | **GW** | 0.1160 | 0.4330 | 0.2947 | 0.3083 | 0.2810 | 0.0118 | 0.0117 | 0.0104 | 0.0099 | 0.0047 | 0.2269 | 0.2556 | 0.2836 | 0.2905 | 0.1504 |
|  | **JW.** | 0.1160 | 0.9100 | 0.9844 | 0.5875 | 0.4381 | 0.0118 | 0.0085 | 0.0086 | 0.0094 | 0.0040 | 0.2269 | 0.3641 | 0.3420 | 0.2445 | 0.1351 |
